# Leukodystrophy-associated *POLR3A* mutations down-regulate the RNA polymerase III transcript and important regulatory RNA *BC200*

**DOI:** 10.1074/jbc.RA118.006271

**Published:** 2019-03-21

**Authors:** Karine Choquet, Diane Forget, Elisabeth Meloche, Marie-Josée Dicaire, Geneviève Bernard, Adeline Vanderver, Raphael Schiffmann, Marc R. Fabian, Martin Teichmann, Benoit Coulombe, Bernard Brais, Claudia L. Kleinman

**Affiliations:** From the a Department of Human Genetics, McGill University, Montréal, Québec H3A 0C7, Canada,; the b Lady Davis Institute for Medical Research, Jewish General Hospital, Montréal, Québec H3T 1E2, Canada,; the c Montréal Neurological Institute, McGill University, Montréal, Québec H3A 2B4, Canada,; the d Translational Proteomics Laboratory, Institut de Recherches Cliniques de Montréal (IRCM), Montréal, Québec H2W 1R7, Canada,; the Departments of m Neurology and Neurosurgery and; e Pediatrics, McGill University, Montréal, Québec H3A 0G4, Canada,; the f Department of Internal Medicine, Division of Medical Genetics, Montréal Children's Hospital, McGill University Health Center, Montréal, Québec H4A 3J1, Canada,; the g Child Health and Human Development Program, and; h MyeliNeuroGene Laboratory, Research Institute, McGill University Health Center, Montréal, Québec H4A 3J1, Canada,; the i Division of Neurology, Children's Hospital of Philadelphia (CHOP), Philadelphia, Pennsylvania 19104,; the j Institute of Metabolic Disease, Baylor Research Institute, Dallas, Texas 75204,; k INSERM U1212–CNRS UMR5320, Université de Bordeaux, Bordeaux, France, and; the l Département de Biochimie et Médecine Moléculaire, Université de Montréal, Montréal, Québec H3T 1J4, Canada

**Keywords:** RNA polymerase III, transfer RNA (tRNA), myelin, CRISPR/Cas, proteomics, brain cytoplasmic 200 RNA (BCYRN1), leukodystrophy, oligodendrocyte, RNA-seq, transcription

## Abstract

RNA polymerase III (Pol III) is an essential enzyme responsible for the synthesis of several small noncoding RNAs, a number of which are involved in mRNA translation. Recessive mutations in *POLR3A*, encoding the largest subunit of Pol III, cause POLR3-related hypomyelinating leukodystrophy (POLR3–HLD), characterized by deficient central nervous system myelination. Identification of the downstream effectors of pathogenic POLR3A mutations has so far been elusive. Here, we used CRISPR-Cas9 to introduce the *POLR3A* mutation c.2554A→G (p.M852V) into human cell lines and assessed its impact on Pol III biogenesis, nuclear import, DNA occupancy, transcription, and protein levels. Transcriptomic profiling uncovered a subset of transcripts vulnerable to Pol III hypofunction, including a global reduction in tRNA levels. The brain cytoplasmic BC200 RNA (*BCYRN1*), involved in translation regulation, was consistently affected in all our cellular models, including patient-derived fibroblasts. Genomic *BC200* deletion in an oligodendroglial cell line led to major transcriptomic and proteomic changes, having a larger impact than those of *POLR3A* mutations. Upon differentiation, mRNA levels of the *MBP* gene, encoding myelin basic protein, were significantly decreased in *POLR3A*-mutant cells. Our findings provide the first evidence for impaired Pol III transcription in cellular models of POLR3–HLD and identify several candidate effectors, including BC200 RNA, having a potential role in oligodendrocyte biology and involvement in the disease.

## Introduction

Mutations in *POLR3A*, *POLR3B*, and *POLR1C,* encoding subunits of the ubiquitous RNA polymerase III (Pol III),^4^ cause the second most common form of childhood-onset hypomyelinating leukodystrophy (HLD) (MIM no. 607694, no. 614381, and no. 616494) (1–3), a type of inherited neurodegenerative disorder characterized by deficient cerebral myelin formation (4). Patients affected with POLR3-related HLD (POLR3–HLD) present in early childhood or adolescence with motor regression and cerebellar features, including ataxia and extra-neurological manifestations, such as hypodontia and/or hypogonadotropic hypogonadism (5). Specific intronic *POLR3A* mutations were also recently linked to hereditary spastic ataxia (6, 7). Histopathological findings in POLR3–HLD patients support a contribution of oligodendrocytes, the myelin-producing cells, as well as neurons, to the disease pathogenesis (5, 8). Nonetheless, the molecular basis of the disease pathophysiology remains poorly understood.

The fact that the central nervous system (CNS) is specifically affected in POLR3–HLD remains puzzling, as most Pol III transcripts are highly abundant and expressed ubiquitously. Indeed, the CNS appears to be particularly vulnerable to impaired Pol III and/or tRNA function, because mutations in a basal Pol III transcription factor also cause a rare cerebellar disorder (9, 10), whereas a growing number of neurological disorders, including several leukodystrophies, are caused by mutations in genes related to tRNA biology (9, 11–21). A notable exception to the ubiquitous expression of Pol III transcripts is BC200 RNA, a primate-specific transcript that is almost exclusively expressed in the brain, with much lower expression in certain other tissues and in various cell lines (22, 23). This ncRNA localizes to neuronal dendrites, where it is thought to regulate local translation (23–25), but its potential involvement in myelination or oligodendrocyte biology has never been investigated.

*POLR3A*, *POLR3B*, and *POLR1C* encode subunits of Pol III, one of the three essential eukaryotic nuclear RNA polymerases. POLR3A and POLR3B are the two largest subunits of Pol III and form the active center of the enzyme (26), whereas POLR1C is a shared subunit of Pol I and Pol III and is thought to serve as a scaffold for the assembly of the enzyme core (27). Pol III synthesizes a diverse group of small ncRNAs, including nuclear-encoded tRNAs (tRNA), 5S ribosomal RNA (rRNA), U6 small nuclear RNA (snRNA), and many others (28). The majority of Pol III transcripts are key players in essential processes such as regulation of transcription, RNA processing, mRNA splicing, and translation (28). Pol III-transcribed genes are broadly grouped into three types based on their promoter elements and associated transcription factors. The type 1 promoter, which is exclusive to 5S rRNA genes, and type 2 promoters (tRNAs and a few others) contain internal elements located within the transcribed sequence (29). A handful of genes that possess type 2 promoters (BC200, 7SL, and vault RNAs) also have upstream promoter elements (30, 31). In contrast, type 3 genes (*U6, RN7SK, RPPH1,* and *RMRP*) exclusively possess upstream promoter elements (29, 32).

Despite its crucial cellular role, the general consequences of Pol III hypofunction in mammalian cells remain obscure, to the point that it is still unknown whether mutations in Pol III subunits impair the transcription of its target genes. Missense and nonsense disease-causing mutations are scattered throughout *POLR3A*, *POLR3B*, and *POLR1C* and are located in multiple functional domains (33). We previously showed that exogenous expression of two POLR1C mutants in HeLa cells impairs Pol III complex assembly, leading to an accumulation of the mutated subunits in the cytoplasm and decreased Pol III binding at most target genes (2). However, whether such a widespread effect on Pol III occupancy is identical for all mutations remains to be established. Of particular interest is the disease-causing *POLR3A* c.2554A→G (p.M852V) mutation, located in the cleft domain of Pol III harboring the template DNA during transcription (26, 33, 34), and more specifically on the bridge helix, which is important for the translocation of the RNA–DNA duplex during transcription (35). As such, it is predicted to directly impair Pol III DNA binding and/or transcription elongation.

In this study, we performed comprehensive profiling of the Pol III transcriptome in human cells carrying the *POLR3A* M852V mutation. We found that a subset of Pol III transcripts is more vulnerable to Pol III hypofunction. In particular, we observed a global decrease in precursor tRNA levels, as well as down-regulation of BC200 RNA in multiple datasets, including POLR3–HLD patient-derived fibroblasts. Knockout (KO) of BC200 in an oligodendroglial-derived cell line results in major transcriptomic and proteomic changes, suggesting an important functional role of BC200 RNA in this cell type. Altogether, our results indicate that tRNAs, BC200 RNA, and its downstream targets may be important effectors of POLR3–HLD pathogenesis.

## Results

### Generation of a cellular model of POLR3–HLD

We first stably expressed FLAG-tagged versions of the wildtype (WT) and mutant (M852V) POLR3A in HeLa cells and performed anti-FLAG affinity purification followed by MS as well as immunofluorescence. POLR3A–M852V pulled down similar levels of Pol III complex subunits compared with the WT and did not accumulate in the cytoplasm (Fig. S1), indicating that this mutation does not impair Pol III complex assembly or nuclear import as did the previously reported *POLR1C* mutations (2). This provides an opportunity to examine the effects of *POLR3A* mutations directly on chromatin occupancy and/or transcription elongation, without the confounding effects of complex assembly or nuclear import deficiencies.

**Figure 1.**
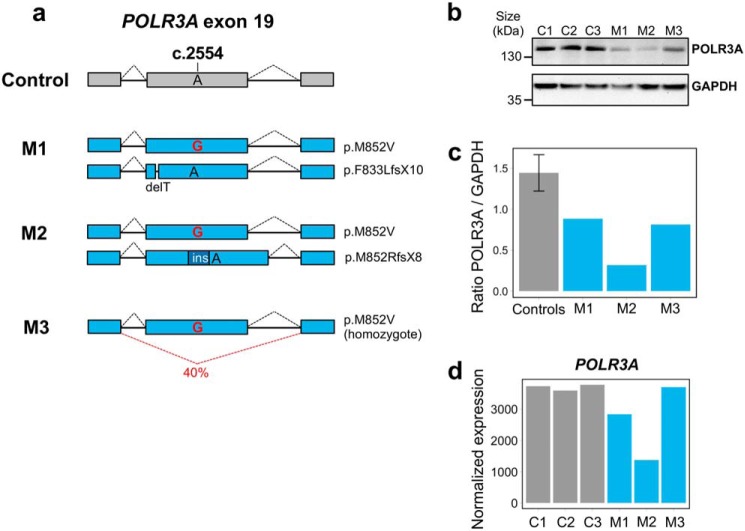
**Characterization of POLR3A mutant clones obtained by CRISPR-Cas9.** *a*, schematic of the genotypes in *POLR3A* mutant clones. Mutants M1 and M2 are compound-heterozygous for the M852V mutation and a deletion/insertion leading to a premature stop codon. The predicted protein change is indicated on the *right*. Mutant M3 is homozygous for the M852V mutation, but this results in partial exon 19 skipping at the mRNA level (see Fig. S2 for details). Exon and intron sizes are not at scale. *b*, POLR3A protein levels in control and mutant clones. GAPDH was used as a loading control. *c*, quantification of POLR3A protein levels normalized to GAPDH levels. The three controls were grouped together. *d*, *POLR3A* mRNA levels in control and mutant clones, quantified by RNA-seq. Mutant M3 has normal levels of *POLR3A* mRNA, suggesting exon 19 skipping does not affect mRNA stability. *ins* indicates insertion.

To explore the transcriptional impact of the *POLR3A* mutations in a cellular model with physiological Pol III levels, we introduced the c.2554A→G (p.M852V) mutation in the endogenous *POLR3A* gene in HEK293 cells using the CRISPR-Cas9 system (36). We obtained one homozygous mutant clone and two compound heterozygous clones that carry the M852V mutation on one allele and an indel causing a frameshift and premature stop codon on the other allele (Fig. 1*a* and Fig. S2*a*). These clones mimic the genotypes observed in POLR3–HLD cases carrying the c.2554A→G (p.M852V) mutation, because four out of the five reported patients that are compound heterozygous for this mutation have a premature stop codon on the other allele (3, 5). This null allele is mostly degraded (Fig. S3) in patient-derived fibroblasts, resulting in decreased levels of POLR3A protein (3). Similarly, POLR3A protein levels were decreased in all three HEK293 mutant clones compared with controls (Fig. 1, *b* and *c*). *POLR3A* mRNA levels were reduced in clones M1 and M2 compared with WT clones but not in the homozygous mutant clone M3 (Fig. 1*d*). Upon inspection of the cDNA, we observed partial (∼40%) in-frame skipping of exon 19, carrying the M852V mutation, in clone M3 (Fig. 1*a* and Fig. S2, *b* and *c*). It was previously shown that in-frame skipping of exons carrying an indel sometimes occurs following CRISPR-Cas9 editing (37), and our observations suggest that this can also happen with point mutations. In sum, we generated three HEK293 clonal cell lines carrying the *POLR3A* M852V mutation and showing reduced levels of POLR3A protein, allowing us to investigate the downstream impact of POLR3A haploinsufficiency.

### POLR3A mutations lead to reduced levels of a subset of its transcripts

**Figure 2.**
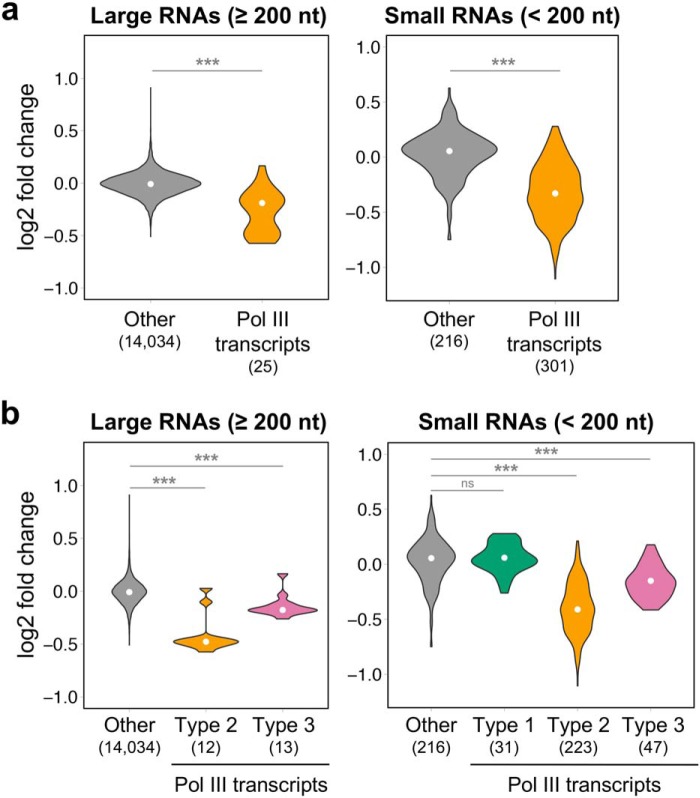
**Pol III transcripts with type 2 internal promoter elements are decreased in POLR3A mutants.** *a*, *violin plots* representing the distribution of expression log2 fold change (mutants/controls) for Pol III transcripts detected in rRNA-depleted RNA-seq (*left*) or small RNA-seq (*right*) compared with all other expressed genes in the respective datasets. *b,* same as *a*, but Pol III transcripts were partitioned into the three types of Pol III promoters. *White points* indicate the median. The number of transcripts in each group are indicated in *parentheses below the graph. nt*, nucleotide; *ns*, non-significant *p*-value. ***, *p* < 0.0001, Wilcoxon rank-sum test.

Pol III occupancy levels measured by ChIP-seq have traditionally been used as a proxy for Pol III transcription levels (38, 39), because measuring Pol III transcripts levels is challenging given that they are strongly structured, chemically modified, and highly repetitive. To assess Pol III occupancy in *POLR3A* mutant cells, we performed ChIP-seq with an antibody against POLR3A in control and mutant clones and observed no significant differences in POLR3A binding at known Pol III target loci (Fig. S4, *a* and *b* and Table S1). ChIP followed by quantitative PCR at three Pol III-transcribed genes also showed comparable POLR3A occupancy in the two groups (Fig. S4*c*). Although a direct correlation generally exists between Pol III occupancy and nascent RNA levels (40), this correlation would be disrupted if the M852V mutation were to affect transcription elongation. Thus, to directly assess transcriptional effects of this mutation, we used two complementary RNA-seq approaches in the three mutant and three control clones, covering the full-size range of Pol III transcripts (70–330 nucleotides). First, we developed a custom small RNA-seq method to properly quantify Pol III transcripts smaller than 200 nucleotides, so as to ensure proper sequencing depth of tRNA precursors (supporting Methods and Figs. S5 and S6). We synthesized exogenous spike-in RNAs to confirm small RNA enrichment (Fig. S6*a*) and focused on precursor tRNAs to (i) overcome the quantification challenges associated with the strong secondary structure and RNA modifications of mature tRNA, and (ii) to more directly assess Pol III transcription levels, without the confounding effects of tRNA stability (Fig. S6, *b* and *c*). In contrast to the drops in coverage expected at highly-modified positions in mature tRNAs (41, 42), we obtained a uniform coverage of tRNA gene bodies (Fig. S6*b*), indicating that most reads originate from precursor tRNA molecules that have not yet acquired post-transcriptional modifications. For Pol III transcripts larger than 200 nucleotides, we performed standard rRNA-depleted RNA-seq. Pol III transcripts showed a global trend toward decreased expression in mutants compared with controls in both RNA-seq datasets (Fig. 2*a*). Furthermore, stratifying according to their promoter type revealed that this decrease was mostly attributable to Pol III transcripts possessing type 2 internal elements, whereas type 3 transcripts were less affected (Fig. 2*b*).

**Figure 3.**
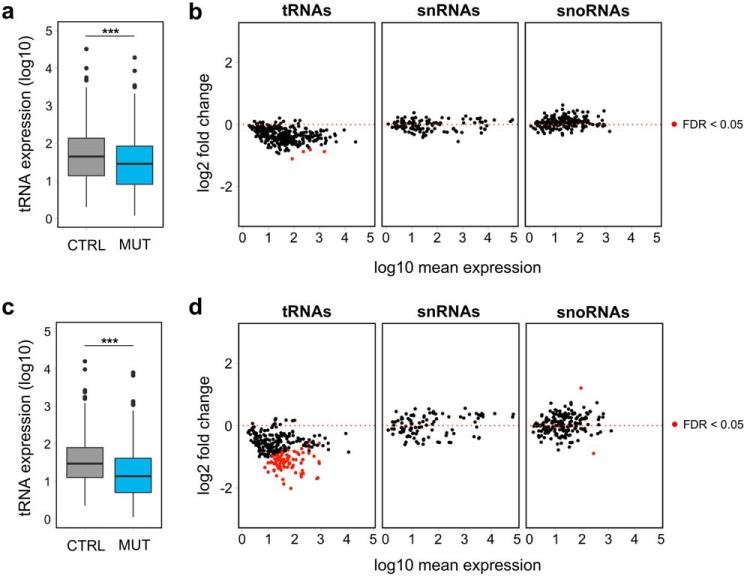
**Differential expression analysis shows decreased tRNA precursor levels in mutants.** *a* and *c*, distribution of normalized expression for all detected tRNAs in the small RNA-seq data, showing a general decrease in global tRNA levels in three mutants (*MUT*) (M1–M3) compared with three controls (*CTRL*) (C1–C3) (*a*) and biological triplicates of mutant M2 and control C3 (*c*). ***, *p* value < 0.0001, Wilcoxon rank-sum test. *b* and *d,* MA plots for all detected tRNAs, snRNAs, and snoRNAs in the small RNA-seq data, showing a general down-regulation of tRNA genes in three mutants (M1–M3) compared with three controls (C1–C3) (*b*) and biological triplicates of mutant M2 and control C3 (*d*). In contrast, snRNAs and snoRNAs are not affected by the POLR3A mutation, consistent with the fact that the majority is not synthesized by Pol III.

**Figure 4.**
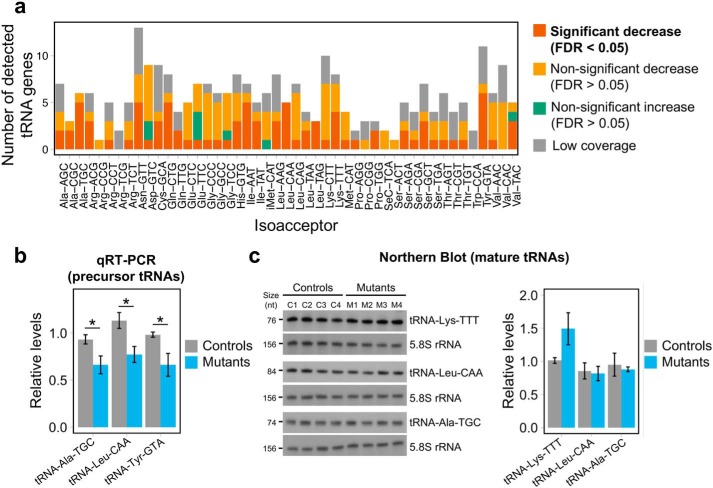
**Global decrease in tRNA levels in *POLR3A* mutants.** *a*, differential expression results from small RNA-seq for detected tRNA genes, categorized by isoacceptor families, showing that affected tRNAs belong to most families. The threshold for low tRNA coverage was mean normalized expression <10. *b*, expression of three tRNA genes measured by qRT-PCR in total RNA from three controls (C1–C3) and three mutants (M1–M3). tRNA gene expression was normalized to the stable genes *SDHA*, *PSMB6*, and *COPS7A* using the ΔΔ*Ct* method. For tRNA–Leu–CAA and tRNA–Tyr–GTA, primers are specific to the precursor form, and primers for tRNA–Ala–TGC do not discriminate between the precursor and mature forms at the sequence level. Groups were compared using a one-tailed Student's *t* test. *c, left*, expression of mature tRNAs measured by Northern blotting in four control (*C1–C4*) and four *POLR3A* mutant (*M1–M4*) HEK293 cell lines. The *top blot* was sequentially probed with BC200 RNA (Fig. 5*c*), tRNA–Lys–TTT, and 5.8S rRNA, which was used as a loading control for both figures. The *middle* and *bottom blots* were sequentially probed with tRNA–Leu–CAA or tRNA–Ala–TGC and 5.8S rRNA as a loading control. Transcript sizes in nucleotides (nt) are indicated on the *left. Right,* quantification of the Northern blotting. tRNA levels were normalized by 5.8S rRNA levels. *, *p* value < 0.05.

Because nuclear-encoded tRNAs represent the majority of transcripts with a type 2 promoter, we examined their expression in the small RNA transcriptome data, consisting mostly of precursor tRNAs. Indeed, we observed a global reduction of precursor tRNA levels in the three mutants compared with controls (*p* < 0.0001, Wilcoxon rank-sum test) (Fig. 3*a*). However, although the majority of expressed precursor tRNAs showed a trend toward a decrease (Fig. 3*b* and Table S2), at the individual gene level only four tRNA genes demonstrated a statistically significant decrease (FDR <0.05), due to the variability in the genotypes of the three mutants. To reduce variability and analyze the effects of the potentially most detrimental *POLR3A* mutations on tRNA expression, we also analyzed biological triplicates of mutant M2, the clone with the lowest *POLR3A* expression (Fig. 1, *c* and *d*). Indeed, the large majority of nuclear-encoded tRNAs showed decreased expression in M2 compared with control C3 (Fig. 3, *c* and *d*), with 77 tRNAs reaching statistical significance at the individual gene level (Fig. 3*d* and Table S3). In contrast, levels of other categories of small RNAs (snRNAs and snoRNAs), the majority of which are not synthesized by Pol III, were unaffected, both in individual clones (Fig. 3*b*) and in replicates of clone M2 (Fig. 3*d*). Decreased tRNAs belonged to most tRNA isoacceptor families (Fig. 4*a*), suggesting a widespread effect on transcription of tRNA genes and arguing against a specific impact on tRNAs cognate for certain codons or amino acids. We next confirmed the decreased precursor tRNA levels by an independent method, measuring the expression levels of three tRNAs by qRT-PCR with primers specific to the precursor form for two of them (Fig. 4*b*). However, the down-regulation of precursor levels did not result in a decrease in levels of three mature tRNAs detectable by Northern blots (Fig. 4*c*), suggesting that the stability of tRNAs is sufficient to overcome the impaired tRNA synthesis in HEK293 cells. Altogether, these results provide the first evidence that a leukodystrophy-causing *POLR3A* mutation causes a global reduction in transcription levels of nuclear-encoded tRNA genes.

**Figure 5.**
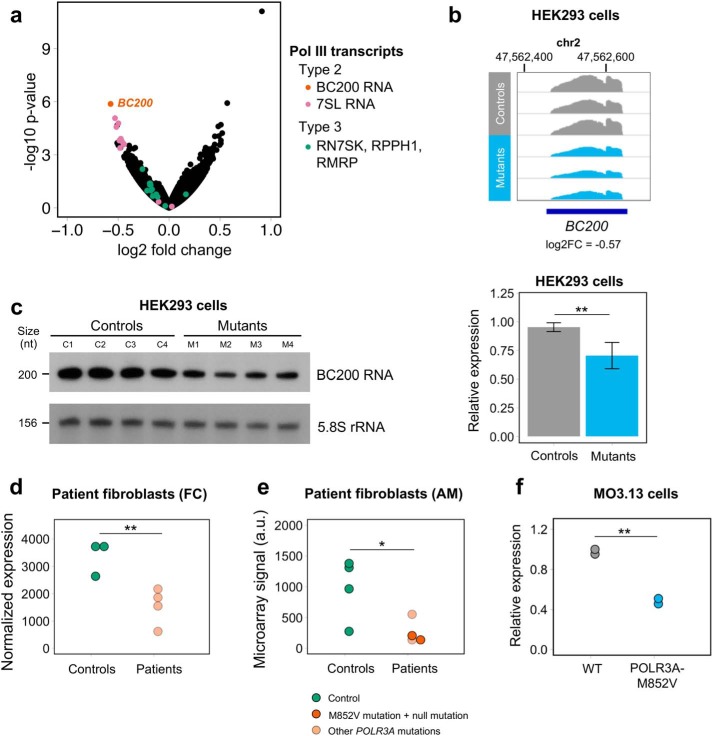
**BC200 RNA is the top down-regulated Pol III transcript in multiple datasets of *POLR3A* mutant cells.** *a*, *volcano plot* representing the results of differential expression analysis between HEK293 controls and mutants using rRNA-depleted RNA-seq (long RNA transcriptome). Expressed genes and pseudogenes encoding Pol III transcripts are shown in distinct colors based on their promoter type. *b,* Integrative Genomics Viewer screenshot of normalized BC200 expression in HEK293 controls and mutants. All samples are represented on the same scale (0–2900). *c*, *left*, expression of BC200 RNA measured by Northern blotting in four control and four *POLR3A* mutant HEK293 cell lines. Transcript sizes in nucleotides (*nt*) are indicated on the *left.* This blot was sequentially probed with BC200 RNA, tRNA–Lys–TTT (Fig. 4*c*), and 5.8S rRNA, which was used as a loading control for both figures. *Right*, quantification of the Northern blotting. BC200 RNA levels were normalized by 5.8S rRNA levels. The two groups were compared with a one-sided Student's *t* test. *d*, RNA-seq BC200 RNA normalized expression in primary fibroblasts from controls and French Canadian (*FC*) POLR3–HLD patients. *e*, microarray signal for a probe targeting the 3′ unique sequence of BC200 RNA in fibroblasts from controls and American (*AM*) POLR3–HLD patients. The two carriers of the M852V mutation are shown in *darker orange*. The two groups were compared with a one-tailed Student's *t* test. *f,* expression of BC200 RNA measured by qRT-PCR in the WT and *POLR3A* mutant MO3.13 cells. BC200 RNA expression was normalized to *PMM1* and *NDUSF2* using the ΔΔ*Ct* method. The two groups were compared with a one-tailed Student's *t* test. *, *p* value < 0.05; **, *p* < 0.01.

We next examined the other transcripts with type 2 internal promoter elements, which include BC200 RNA, 7SL RNAs, and vault RNAs (28, 29, 31). *BC200* (also called *BCYRN1*) and genes encoding 7SL RNA were among the most significantly down-regulated genes in the large RNA transcriptome (Fig. 5*a* and Table S4). In contrast, vault RNA levels were comparable across both groups, making them the only transcripts with type 2 internal elements that were not decreased in mutants, which could be due to their vault RNA-specific distal regulatory sequences (31). Consistent with a selective effect on type 2 transcripts, none of the genes coding for type 1 (5S rRNA) or type 3 transcripts (U6 RNAs, Y RNAs, RPPH1, RMRP, and RN7SK) displayed significant differences between control and mutant samples, although some showed a small trend toward decreased expression in mutants (Fig. S7*a* and Tables S2–S4). Taken together, these results strongly suggest that transcripts possessing type 2 internal promoter elements are the most vulnerable to the studied disease-causing *POLR3A* mutation.

### BC200 RNA is down-regulated in patient-derived fibroblasts

Among the large RNA transcriptome, BC200 RNA, the only Pol III transcript observed to have a predominant CNS expression, was the most down-regulated transcript in HEK293 mutant clones (Fig. 5, *a* and *b*), suggesting a particular sensitivity to Pol III hypofunction. To validate this finding, we performed Northern blotting for BC200 RNA in four controls and four *POLR3A* mutants (M1 to M3 and an additional cell line (M4) carrying the F849L mutation in *POLR3A* (see supporting Methods)). This confirmed that BC200 RNA levels are significantly decreased in mutants compared with controls (Fig. 5*c*). To determine whether this transcript was also affected in patient cells, we examined two independent datasets of patient-derived fibroblasts. First, in RNA-seq from four French Canadian *POLR3A* patients (see genotypes in Table S5), BC200 RNA was the top affected Pol III transcript in patients compared with controls (Fig. 5*d*, Fig. S7*b*, and Table S6), although not reaching statistical significance after multiple testing correction (*p* = 0.004, FDR = 0.102). Other large Pol III transcripts did not display significant differences between patients and controls (Fig. S7*b*). Second, using a custom microarray for all known Pol III transcripts in four American *POLR3A* cases (including two cases carrying the M852V mutation, see Table S5), we found that BC200 RNA displayed a significant reduction in patients compared with controls (Fig. 5*e*). Altogether, we show that BC200 RNA is the top down-regulated large Pol III transcript in three separate datasets of *POLR3A* mutant cells, including carriers of several distinct mutations, emphasizing its vulnerability to Pol III hypofunction and suggesting a possible role in the pathophysiology of POLR3–HLD.

### Absence of BC200 RNA leads to important changes in the transcriptome and proteome of MO3.13 cells

**Figure 6.**
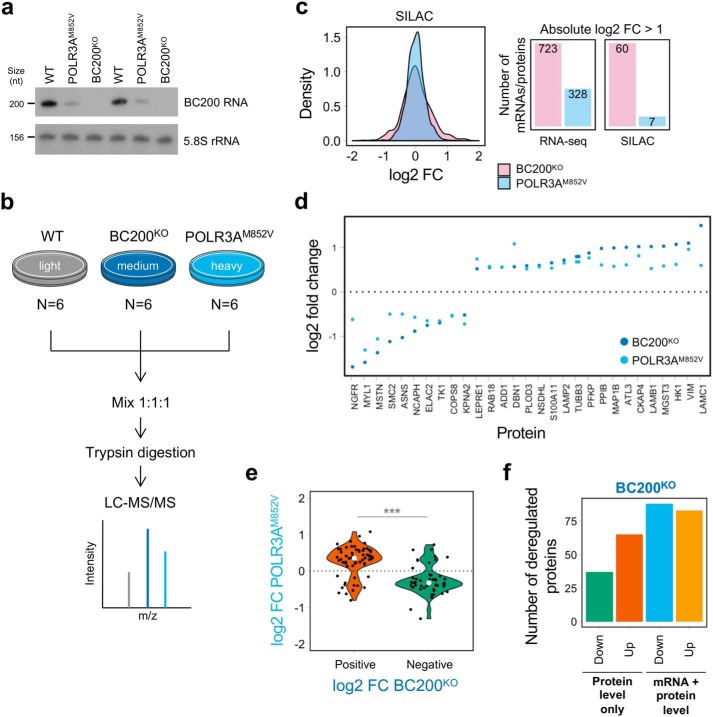
**BC200 KO causes important changes at the proteome level in MO3.13 cells.** *a*, Northern blotting showing decreased expression of BC200 RNA in POLR3A^M852V^ cells compared with WT and complete absence of BC200 RNA in BC200^KO^ cells. 5.8S rRNA was used as a loading control. The experiment was performed for biological duplicates. Transcript sizes in nucleotides (*nt*) are indicated on the *left. b*, overview of the SILAC protocol. *Light* refers to unlabeled amino acids; *medium* to Lys-4 and Arg-6, and *heavy* to Lys-8 and Arg-10. *c*, *left*, distribution of the SILAC log2 fold change for POLR3A^M852V^/WT and BC200^KO^/WT. *Right*, number of mRNAs/proteins with absolute log2 fold change of >1 in POLR3A^M852V^/WT and BC200^KO^/WT. More proteins have high fold changes in BC200^KO^ (*p* value = 3.02 × 10^−8^, two-sample Kolmogorov-Smirnov test). *d*, log2 fold change for proteins that are differentially abundant (FDR <0.05, absolute log2 fold change >0.5) and move in the same direction in both mutant conditions compared with WT. *e*, distribution of log2 fold change in POLR3A^M852V^ for proteins that showed statistically significant differences (FDR <0.05) in both mutant conditions and had a significant fold change in BC200^KO^ (log2 FC >0.5). Proteins tend to change in the same direction in both conditions but with a lower effect size in POLR3A^M852V^ compared with BC200^KO^. ***, *p* < 0.001, Wilcoxon rank-sum test. *f*, number of mRNA/protein pairs where the protein shows substantially greater fold changes than the mRNA in BC200^KO^ *versus* WT (protein level only) and where the protein and mRNA have similar fold changes (mRNA + protein level). Each category is further separated into *up*- and *down*-regulated proteins in BC200^KO^. *FC* indicates fold change.

Given the expression of BC200 RNA in the CNS and its consistent down-regulation in *POLR3A* mutant cells and patient-derived fibroblasts, we next investigated the effects of decreasing BC200 RNA expression. Because oligodendrocytes are likely to be one of the main dysfunctional cell types in POLR3–HLD (5, 8) for their role in myelination, we opted to use the MO3.13 cell line, which displays features of oligodendrocyte precursor cells (OPC) and is one of only two existing human oligodendroglial immortalized cell lines (43, 44.) First, we used CRISPR-Cas9 to delete the entire BC200 gene in MO3.13 cells (Fig. S8, *a–c*), resulting in absent expression (Fig. S8*d*). Next, using the same approach as above, we generated MO3.13 cells carrying the *POLR3A* c.2554A→G (p.M852V) mutation in compound heterozygosity with a null allele (Fig. S8*e*). We verified that *POLR3A* mutant MO3.13 cells recapitulate the effects observed in HEK293 cells. Indeed, they display reduced levels of POLR3A protein (Fig. S8*f*). They also show significantly decreased levels of BC200 RNA (Fig. 5*f* and Figs. S8*d* and S9). Consistent with our above results, type 3 Pol III transcripts *RN7SK*, *RPPH1*, and *RMRP* were unaffected in POLR3A^M852V^ cells (Fig. S9 and Table S7). However, contrary to what we observed in HEK293 cells, 7SL RNA levels were not decreased in mutant MO3.13 cells (Fig. S9), suggesting cell type–specific effects that could be related to upstream regulatory sequences specific to 7SL genes (31). In sum, we obtained two mutant cell lines with decreased levels of BC200 RNA (∼50% reduction in POLR3A^M852V^ and absent in BC200^KO^), which was confirmed by Northern blotting (Fig. 6*a*).

Given the potential role of BC200 RNA in mRNA stability, transport, and translation regulation (25, 45, 46), we investigated both steady-state mRNA and protein levels in these cell lines. To complement the RNA-seq data, we applied quantitative MS by stable isotope labeling of amino acids in culture (SILAC) (Fig. 6*b*), detecting a total of 1,272 proteins. We found that, compared with WT cells, BC200 KO had a more potent effect on the transcriptome and proteome than the *POLR3A* M852V mutation did, leading to a higher number of dysregulated mRNAs and proteins and to more of them showing a large effect size (*p* value < 0.0001, two-sample Kolmogorov-Smirnov test) (Fig. 6*c* and Fig. S10, *a* and *b*). In particular, more proteins were up-regulated than down-regulated in BC200^KO^ cells (skewness = 0.566, *p* value = 5.29 × 10^−14^, D'Agostino test of skewness) (Fig. 6*c* and Fig. S10*a*). These observations point to a clear functional role for BC200 RNA in MO3.13 cells, because its inactivation leads to significant changes for more than 20% of detected proteins (276/1,272) and expressed mRNAs (3,165/13,281) (Tables S7 and S8). Gene Ontology analysis showed an over-representation of proteins located in the plasma membrane, endoplasmic reticulum, and cytoskeleton among the up-regulated proteins. In contrast, down-regulated proteins were enriched for ones involved in the G_1_/S transition, including members of the condensin and minichromosome maintenance complexes, suggesting slower cell growth or cell cycle defects.

For the remainder of our analyses, we focused on mRNA/protein pairs that were properly detected in both the RNA-seq and SILAC datasets (*n* = 1,200) (Fig. S10*c* and Table S9) and searched for common effects in BC200^KO^ and POLR3A^M852V^. Although only 29 proteins were consistently significantly dysregulated (log2 fold change >0.5) in both mutants (Fig. 6*d*), we observed that proteins affected in BC200^KO^ (FDR <0.05, log2 fold change >0.5) tended to be affected in the same direction if they were also significant in POLR3A^M852V^ (FDR <0.05), albeit with a lower fold change (Fig. 6*e*). We also observed a similar effect at the mRNA level (Fig. S10*d*). This suggests that the changes in mRNA and protein levels observed in POLR3A mutant cells may be partially explained by reduced expression of BC200 RNA and further support a contribution of this ncRNA to POLR3–HLD pathogenesis.

Because BC200 RNA has been repeatedly implicated in the regulation of translation *in vitro* (25, 47, 48), we next asked whether the observed changes in protein levels in BC200^KO^ cells were the result of deregulation at the mRNA or at the protein level. Thus, we measured the difference between the SILAC log2 fold change and the RNA-seq log2 fold change for each mRNA/protein pair. We identified 48 proteins (17.4% of differentially abundant proteins) that were significantly increased or decreased in BC200^KO^ compared with WT but that did not display significant differences at the mRNA level (Fig. S11), implying protein-level regulation. Furthermore, we found an additional 54 proteins (19.6% of differentially abundant proteins) that had substantially greater fold changes compared with their mRNA counterparts (Fig. S11). Thus, although the majority of deregulated proteins (63.0%) have similar fold changes in SILAC and RNA-seq, suggesting that their regulation occurs at the transcriptional/mRNA level, the remainder (37.0%) appears to undergo changes at the protein level only as a result of BC200 KO (Fig. 6*f*), perhaps through the direct action of this ncRNA on translation initiation (25). Interestingly, there is a larger number of up-regulated proteins (*n* = 65) than down-regulated ones (*n* = 37) in this category (Fig. 6*f*), which is consistent with the putative role of BC200 as a negative regulator of translation (47). Altogether, these analyses highlight candidate proteins that could be directly regulated by BC200 RNA at the translational level, whereas others are directly or indirectly modulated at the transcriptional level.

### POLR3A mutations lead to decreased expression of MBP

**Figure 7.**
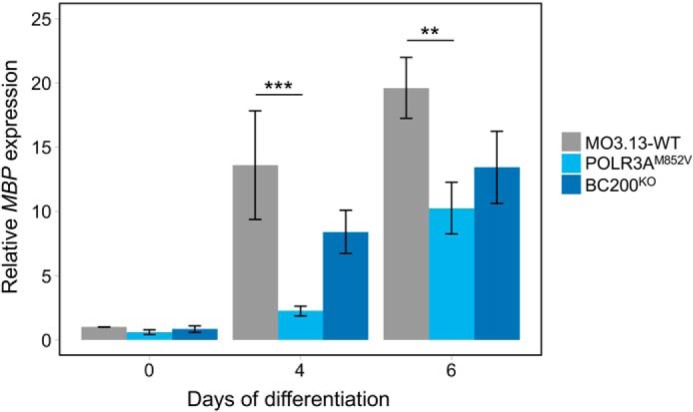
**Decreased *MBP* expression in POLR3A^M852V^ cells during MO3.13 differentiation.** MO3.13-WT, POLR3A^M852V^, and BC200^KO^ cells were cultured in serum-free medium supplemented with 100 nm PMA to induce their differentiation. Cells were collected, and RNA was extracted after 0 (untreated), 4, and 6 days of differentiation, and *MBP* mRNA expression was measured by qRT-PCR and normalized to the stable genes *PMM1* and *NDUFS2* using the ΔΔ*Ct* method. Data are represented as mean ± S.E. of four independent experiments. Conditions were compared with a two-way ANOVA and Tukey's multiple comparisons test. ***, *p* < 0.001; **, *p* < 0.01.

Because impaired myelin formation is one of the main pathological features of POLR3–HLD (5), we next assessed the impact of *POLR3A* mutations and BC200 KO on the differentiation of MO3.13 cells. MO3.13 cells, which exhibit features of OPCs, can be differentiated into more mature oligodendrocyte-like cells that start expressing *MBP*, encoding one of the major proteins of CNS myelin (43, 49). *MBP* mRNA levels were significantly decreased in POLR3A^M852V^ cells compared with WT cells after 4 and 6 days of differentiation (Fig. 7), showing that *POLR3A* mutations impair the expression of an important marker of mature oligodendrocytes. Furthermore, BC200^KO^ cells also showed a trend toward decreased *MBP* expression compared with WT cells, although the effect was milder than in POLR3A^M852V^ cells (Fig. 7). This could indicate that decreased BC200 RNA expression in POLR3A^M852V^ cells contributes to impaired differentiation but that other factors, such as altered tRNA levels, also play a role in this phenotype. Nevertheless, these results provide the first evidence supporting oligodendrocyte differentiation and/or *MBP* mRNA expression alteration in a cellular model of POLR3–HLD.

## Discussion

Mutations in genes encoding Pol III subunits cause POLR3–HLD, a devastating neurodegenerative disorder. However, the extent to which Pol III mutations affect expression of Pol III transcripts remains unclear. Identification of specific Pol III transcripts that are deregulated in POLR3–HLD could provide clues to the downstream disease mechanisms. Herein, we combined CRISPR-Cas9 gene editing and transcriptomic profiling in human cells to uncover Pol III transcripts that are most vulnerable to POLR3–HLD disease-causing mutations.

We found that the *POLR3A* M852V mutation leads to significantly decreased expression of some but not all Pol III transcripts. The common feature of these transcripts is their possession of type 2 Pol III promoter elements (29), suggesting a promoter-specific vulnerability to Pol III dysfunction, although not all members of this group were down-regulated (*i.e.* vault RNAs, 7SL RNAs in MO3.13 cells). This type of promoter is composed of control elements located within the transcribed sequence, called A- and B-boxes (32). In addition, expression of BC200, 7SL, and vault RNAs also requires various upstream promoter and/or enhancer elements (30, 31). In contrast, type 3 promoters are entirely located upstream of the transcription start site and include proximal and distal elements that are highly similar to the ones controlling the transcription of other snRNA genes by Pol II and are recognized by the same transcription factors (32, 33). Considering these similarities, one possibility is that Pol II can compensate for decreased Pol III transcription at type 3 genes, thus stabilizing the levels of these transcripts in *POLR3A* mutants. At least one type 3 gene, *RPPH1,* has been shown to be occupied in equivalent amounts by Pol III and Pol II (50). Alternatively, the internal nature of type 2 promoters could be particularly detrimental to a mutated Pol III. A- and B-boxes are bound by the transcription factor TFIIIC for transcription initiation, but TFIIIC also presumably acts as a block on transcription that must be displaced during elongation, possibly through conformational changes induced by the elongating Pol III (51, 52). Thus, impaired elongation due to *POLR3A* mutations could have a more detrimental effect on type 2 genes, compared with type 3 genes where this physical obstacle to transcription is not present. It is important to note that type 3 Pol III transcripts are not completely resistant to the *POLR3A* M852V mutation, but their down-regulation appears to require a higher degree of Pol III dysfunction. For example, RN7SK and RPPH1 have decreased expressions in mutant M2 only, which also showed the lowest expression of BC200 RNA and tRNAs, suggesting that it has the worst level of preserved Pol III function. This indicates that Pol III transcripts have different sensitivities to POLR3A haploinsufficiency. Furthermore, mutations in *BRF1*, encoding a subunit of TFIIIB specific to type 1 and type 2 promoters, cause a cerebellar–facial–dental syndrome (MIM no. 616202) with significant phenotype overlap with POLR3–HLD (9, 10), thus further suggesting that type 2 transcripts may be of particular importance for disease pathogenesis.

Mature tRNA levels are notoriously difficult to quantify by RNA-seq. Recently, several protocols have been published to overcome the challenges associated with next-generation sequencing of mature tRNAs (41, 42, 53, 54). Alternatively, standard RNA-seq has been used to quantify tRNA precursors and to obtain a more direct assessment of Pol III transcription levels (55). Here, we optimized this approach by enriching for small transcripts, allowing for greater coverage of tRNA genes. We obtained a uniform coverage of tRNA gene bodies, without the significant drops in coverage at highly modified bases in mature tRNAs observed in previous studies, confirming that we are enriching for precursor tRNAs. Consistent with the global, statistically significant but moderate reduction in precursor tRNA levels observed by small RNA-seq and qRT-PCR, Northern blotting for three mature tRNAs did not uncover decreased levels in mutants compared with controls. It is possible that the defective tRNA synthesis observed in HEK293 cells, not sufficient to result in reduced mature tRNA levels in this cell model, would affect these mature tRNA levels in cells that are involved in the pathogenesis of the disease. One of the current leading hypotheses to explain POLR3–HLD pathogenesis is that Pol III hypofunction impairs the transcription of certain tRNAs that are essential at specific developmental times for the synthesis of proteins involved in CNS myelin development (2, 11). In our study, down-regulated tRNA precursors belonged to most tRNA isoacceptor families, and we did not observe a selective effect on certain tRNAs, such as the ones that show higher expression in the brain compared with other tissues (56) or the ones that correspond to tRNA aminoacyl synthetases mutated in other HLDs (12, 14, 15, 17). Additional experiments will be required to determine whether the observed decreased tRNA synthesis levels are sufficient to negatively affect mRNA translation *in vivo* during development. It is possible that reduced tRNA transcription only has a detrimental effect on mature tRNA levels and mRNA translation at times when the need for newly synthesized proteins is very high, such as during myelinogenesis (57), and remains inconsequential in other spatio-temporal contexts.

Assessing the extent to which the transcriptional consequences of the *POLR3A* mutations identified here resemble expression dysregulation in patients is challenging, due to the limited availability of relevant tissue and the confounding effects of cellular composition associated with a demyelinating phenotype. The affected transcripts in our *POLR3A* mutant cells partially overlap with the ones that were decreased in the blood of patients with an atypical Pol III-related disease without myelin abnormalities, caused by partial skipping of *POLR3A* exon 14 (58). The authors also observed significantly decreased expression of 7SL RNA and a small number of tRNAs by RNA-seq. In another study, mutations in *POLR3K* were recently identified in two patients with leukodystrophy. By qRT-PCR, the authors found that one tRNA, 7SL RNA, and 5S rRNA were significantly decreased in fibroblasts from both patients compared with controls (59). Despite the different Pol III subunit affected, the different cell type assessed, and the difference in techniques used, the same transcripts (tRNAs and 7SL) appear to be vulnerable to Pol III dysfunction in all three studies, further strengthening our results.

Among differentially expressed transcripts, BC200 RNA stands out because of its high expression in the brain and the consistent decreased expression in all cellular models carrying different *POLR3A* mutations. Although BC200 RNA was originally exclusively detected in the brain, recent data indicate that it is also expressed in various primary and immortalized human cell lines (22), albeit at lower levels than in the brain. This is consistent with our own detection of BC200 RNA expression in fibroblasts and HEK293 cells. Despite being a primate-specific transcript, BC200 RNA does have a functional analog in rodents, Bc1 RNA, which is of different evolutionary origin but is also synthesized by Pol III and displays similar expression pattern, dendritic localization, and function (23). Bc1 KO in mouse does not lead to hypomyelination or impaired motor function (60). However, considering the documented difficulties of recapitulating human myelin disorders in mice, including by our group in a POLR3–HLD mouse model (61), the absence of a relevant phenotype in Bc1 KO mice is not sufficient to exclude BC200 as one of the potential mediators of the disease.

In the brain, BC200 and Bc1 RNAs are thought to regulate local translation in neuronal dendrites by repressing the function of translation initiation factors eIF4A and eIF4B (25). In human cell lines, BC200 RNA was also found to interact with different proteins to regulate alternative splicing (62), mRNA stability (45), and translation in p-bodies (63), implying that its role goes beyond the analogies drawn from studying Bc1 RNA. In the oligodendroglial MO3.13 cells, we found that BC200 KO led to alterations in steady-state protein levels that were partially recapitulated in *POLR3A* mutant cells, suggesting a dose-dependent effect of decreased BC200 RNA expression. Interestingly, approximately one-third of the deregulated proteins in BC200^KO^ cells appeared to be regulated at the translational or post-translational level, with almost twice as many up-regulated than down-regulated proteins. This suggests a scenario where loss of BC200 RNA lifts its repressive action on eIF4A and eIF4B for certain mRNAs, thus allowing their increased translation. Although BC200 expression was not detected in the white matter of an adult human brain using radioactive *in situ* hybridization (23), it could be expressed in cells of the oligodendroglial lineage earlier in development. The important mRNA and protein changes observed in BC200^KO^ cells suggest that this ncRNA plays a role in the OPC-like MO3.13 cells. Nonetheless, considering that BC200 RNA is up-regulated and essential for cell growth in several cancers (22) and that the MO3.13 cell line was established from a tumor (43), we cannot exclude that its expression or function is specific to this cell line. Thus, validation of our results in primary OPCs or oligodendrocytes would represent an important step to confirm the potential involvement of BC200 RNA in oligodendrocyte function and myelination. Furthermore, although not the main focus of this study, cerebellar atrophy is a major feature of POLR3–HLD and the recently described Pol III-related spastic ataxias (6, 7). Therefore, the function of BC200 RNA in dendrites could also contribute to the loss of cerebellar neurons observed in POLR3–HLD cases, considering the massive dendritic arborization of cerebellar Purkinje cells (64).

We show that *POLR3A* mutations, and to a lesser extent BC200 KO, have a detrimental impact on *MBP* mRNA expression during differentiation of MO3.13 cells. If confirmed *in vivo*, these data would suggest a possible disease mechanism, where impaired differentiation or decreased *MBP* expression as a result of *POLR3A* mutations could lead to a shortage of MBP in compact myelin and to the hypomyelination observed in POLR3–HLD. In addition, the more severe effect observed in POLR3A^M852V^ compared with BC200^KO^ cells indicates that POLR3–HLD is likely the result of impaired expression of several transcripts, including BC200 RNA and tRNAs, which all contribute to the observed phenotypes.

In conclusion, our study shows for the first time that a disease-causing POLR3–HLD mutation causes selective alterations in Pol III transcript levels and that BC200 RNA may play a key role in the pathophysiology of POLR3–HLD. We also establish the first link between *POLR3A* mutations and decreased expression of *MBP*, an important oligodendrocyte marker. Finally, our data pinpoint several Pol III transcripts and candidate downstream targets of BC200 RNA, which can be further investigated for their role in oligodendrocytes, their involvement in the disease, and their potential as future therapeutic targets.

## Materials and methods

### Cell lines

Stable HeLa cell lines expressing FLAG-tagged POLR3A variants (WT or M852V) were produced by transfection with Lipofectamine according to the manufacturer's instructions. MO3.13 cells were purchased from Cedarlane CELLutions Biosystems (no. CLU301). Unless otherwise specified, all cells were grown in 1× DMEM (Wisent no. 319-005-CL) supplemented with 10% fetal bovine serum (Wisent no. 080-150) and 1% penicillin–streptomycin–l-glutamine (Wisent no. 450-202-EL). For differentiation of MO3.13 cells, cells were switched to serum-free 1× DMEM supplemented with 100 nm phorbol 12-myristate 13-acetate (PMA), as described previously (43), and collected after 4 or 6 days. All patients who donated fibroblasts for this study signed an informed consent form approved by the institutional ethics committees of the McGill University Health Center Research Ethics Board (11-105-PED) or the Children's National Medical Center, Washington, D. C. This study abides by the Declaration of Helsinki principles.

### Experiments in FLAG-tagged cell lines

Affinity purification, MS, and immunofluorescence in cell lines expressing FLAG-tagged POLR3A subunits were performed as described previously (2, 61).

### CRISPR-Cas9 gene editing

POLR3A mutant cell lines carrying the c.2554A→G (p.M852V) or c.2547C→G (p.F849L) mutations were generated using CRISPR-Cas9 coupled with homology-directed repair, as described previously (36). *BC200* KO cell lines were generated using an approach adapted from Ref. 62, with dual sgRNAs targeting upstream and downstream of the *BC200* gene (Fig. S8*a*). Detailed methods are described in the supporting Methods.

### RT-PCR

For RT-PCR, 1 μg of RNA was reverse-transcribed using the Superscript III reverse transcriptase (Thermo Fisher Scientific). For detection of the M852V mutation, exons 17–21 of *POLR3A* were amplified (see Table S10 for primers) and Sanger-sequenced (McGill University and Génome Québec Innovation Centre).

### Western blotting

Cells were homogenized in lysis buffer (10 mm Tris-HCl, pH 7.5, 150 mm NaCl, 1 mm EDTA, 1% Triton X-100, and protease inhibitors (Roche Applied Science)) and incubated on ice for 30 min, followed by centrifugation at 14,000 rpm for 10 min and collection of the supernatant. Protein concentration was determined using DC^TM^ protein assay (Bio-Rad). Protein samples were separated onto a 4–12% NuPAGE BisTris gel (Thermo Fisher Scientific) and transferred onto a nitrocellulose membrane (Bio-Rad). Immunoblots were probed with anti-POLR3A (Abcam no. ab96328), anti-GAPDH (GeneTex no. GTX627408), and anti-actin (Abcam no. ab3280).

### Small RNA and tRNA precursor sequencing

Detailed protocols and data analysis are described in supporting Methods. Briefly, small RNAs (<200 nucleotides) were enriched from total RNA using Qiagen miRNeasy (no. 217004), directly followed by random priming, cDNA synthesis, and next-generation sequencing. To monitor the level of small RNA enrichment in each sample, we synthesized three spike-in RNAs (70, 94, and 250 nucleotides) and added them at the beginning of the procedure. Libraries were prepared with the KAPA-stranded RNA-seq library preparation and sequenced on an Illumina HiSeq 2500 with 100-bp single-end reads at the McGill University and Génome Québec Innovation Centre, resulting in an average of 25 million reads/sample. Quality control and trimming were performed as described previously (65). Trimmed reads were aligned to the reference genome hg19 using STAR version 2.3.0e (66), including reads mapping to up to 100 locations. Bigwig tracks were produced using BEDtools (67) and UCSC tools and normalized to the total number of mapped reads. Expression levels were estimated with featureCounts (68) version 1.5.0 using exonic reads in three successive runs with different parameters to treat multimapping reads (see supporting Methods).

### RNA sequencing

Total RNA was extracted using miRNeasy (Qiagen) or TRIzol (Thermo Fisher Scientific), treated with the RNase-free DNase set (Qiagen), and quantified on Nanodrop. RNA Integrity Number (RIN) was assessed on an Agilent Bioanalyzer and was routinely above 8. RNA extractions were submitted to Illumina TruSeq rRNA-depleted stranded library preparation (HEK293 cells and patient fibroblasts) or NEBNext rRNA-depleted stranded library preparation (MO3.13 cells). HEK293 libraries were sequenced on Illumina HiSeq 2500 with 125-bp paired-end reads at an average of 42 million reads/sample. Fibroblast libraries were sequenced on Illumina HiSeq 2000 with 50-bp paired-end reads at an average of 60 million reads/sample. MO3.13 libraries were sequenced on an Illumina HiSeq 4000 with 100-bp paired-end reads at an average of 61 million reads/sample. Quality control and trimming were performed as described previously (65). Trimmed reads were aligned to the reference genome hg19 using STAR version 2.3.0e (66), eliminating reads mapping to more than 10 locations. Bigwig tracks were produced using BEDtools (67) and UCSC tools and normalized to the total number of mapped reads. Expression levels were estimated with featureCounts (68) version 1.5.0 using exonic reads and normalized using DESeq2 (69). Differential expression analysis between control and mutant/patient groups was performed with DESeq2 for genes in the Ensembl version 75 annotation (69). Genes were considered differentially expressed if they had an adjusted *p* < 0.05 and a mean normalized expression of >100. Because Pol III transcripts are highly repetitive, alignment and subsequent analyses were repeated with the inclusion of all multimapping reads to confirm consistency in results. Only results that replicated when using either uniquely mapped or multimapping reads were reported.

### ChIP-seq and ChIP-qPCR

WT or *POLR3A*-mutated cells were cultured to 80% confluence and cross-linked with 1% formaldehyde directly in the cell medium for 5 min followed by a 5-min quenching in 125 mm glycine. For ChIP experiments, nuclei from 6 × 10^6^ cells were lysed and resuspended in sonication buffer (10 mm Tris-HCl, pH 8, 140 mm NaCl, 1 mm EDTA, 0.5 mm EGTA, 0.5% Triton, 0.5% SDS, and protease inhibitors). Chromatin was prepared by sonicating the nuclei in a Covaris E-Series E22 for 8 min at Duty 2. This generated chromatin fragments of 500 bp on average. Sonicated chromatin was immunoprecipitated using rabbit POLR3A antibody (Abcam no. ab96328) bound to magnetic beads (protein A and protein G Dynabeads) (Sigma) for 4 h. The eluates were loaded to a PCR purification kit (Qiagen). Sequencing libraries were prepared from input and ChIP eluates using the TruSeq DNA library preparation kit and sequenced on an Illumina HiSeq 2000 with 50-bp paired-end reads at an average of 18 million reads/sample for input libraries and 56 million reads/sample for ChIP libraries. Quality control and trimming were performed as described previously (2). Trimmed reads were aligned to the reference genome hg19 using bowtie2 version 2.2.9 (70). Bigwig tracks were produced using BEDtools (67) and UCSC tools and normalized to the total number of mapped reads. Reads mapping to Pol III target genes were counted with featureCounts (68) version 1.5.0. Data were normalized to the median of total read counts, and Pol III occupancy scores were calculated as the log2(IP_read_count_/input_read_count_), where IP is immunoprecipitation, as described in Orioli *et al.* (40), for bins including the annotated Pol III-transcribed gene body ±150 bp. qPCRs were performed on input and ChIP eluates as described previously (61), using primers indicated in Table S10.

### qRT-PCR

Total RNA was extracted using miRNeasy (Qiagen), treated with the RNase-free DNase set (Qiagen), and quantified on Nanodrop. RIN was assessed on an Agilent Bioanalyzer and was routinely above 8. For qRT-PCR of tRNAs, BC200 RNA and *MBP* mRNA, 1 μg of RNA was reverse-transcribed using the Superscript III reverse transcriptase (Thermo Fisher Scientific) according to the manufacturer's instructions. Real-time PCR was performed in technical duplicates with a 1:25 dilution (tRNAs and BC200 RNA) or 1:2 dilution (*MBP*) using the SYBR GreenER^TM^ qPCR SuperMix (Thermo Fisher Scientific) on a Bio-Rad CFX96. The ΔΔ*Ct* method was used to calculate relative RNA expression, with normalization to genes *PSMB6*, *SDHA*, and *COPS7A* (HEK293 cells) or *NDUSF2* and *PMM1* (MO3.13 cells). All primers used are indicated in Table S10.

### Northern blots

Total RNA was extracted using miRNeasy (Qiagen), treated with the RNase-free DNase set (Qiagen), and quantified on Nanodrop. RIN was assessed on an Agilent Bioanalyzer and was routinely above 8. RNA samples (3 μg) were separated on a 7 m urea denaturing 8% PAGE and transferred to positively charged nylon transfer membranes (GE Healthcare). The resulting blots were hybridized with ^32^P-5′ end–labeled probes detecting BC200 RNA, tRNA–Ala–TGC, tRNA–Lys–TTT, tRNA–Leu–CAA and 5.8S rRNA in ExpressHyb hybridization solution (TaKaRa). All probes used are indicated in Table S10 and are complementary to the last 24 nucleotides in 3′ of mature tRNAs, including the CCA trinucleotide. 5.8S rRNA was used for normalization of BC200 RNA and tRNA levels.

### SILAC

Detailed reagents, protocols, and data analysis are included in the supporting Methods. Briefly, cells were grown in DMEM for SILAC (Thermo Fisher Scientific, no. 88364) supplemented with 10% dialyzed fetal bovine serum (Thermo Fisher Scientific, no. A3382001) and light, medium, or heavy amino acids for MO3.13 WT, BC200^KO^, and POLR3A^M852V^, respectively (Fig. 6*b*). For each of the six independent replicate experiments, equal protein quantities from the three conditions were mixed. Samples were digested with trypsin and analyzed via LC-MS/MS. Data analysis was performed with MaxQuant (71) version 1.6.0.16 and Perseus version 1.5.6.0.

## Author contributions

K. C., G. B., A. V., R. S., M. T., B. C., B. B., and C. L. K. conceptualization; K. C. and D. F. data curation; K. C., D. F., E. M., M. T., and C. L. K. formal analysis; K. C., D. F., E. M., and M.-J. D. validation; K. C., D. F., E. M., M.-J. D., M. R. F., B. B., and C. L. K. investigation; K. C., D. F., M. R. F., and C. L. K. methodology; K. C., B. B., and C. L. K. writing-original draft; K. C. project administration; K. C., D. F., E. M., M.-J. D., G. B., A. V., R. S., M. R. F., M. T., B. C., B. B., and C. L. K. writing-review and editing; G. B., B. C., B. B., and C. L. K. funding acquisition; B. C., B. B., and C. L. K. supervision; C. L. K. resources.

## Supplementary Material

Supporting Information

## Footnotes

4 The abbreviations used are:

Pol III: RNA polymerase III
HLD: hypomyelinating leukodystrophy
CNS: central nervous system
PMA: phorbol 12-myristate 13-acetate
FDR: false discovery rate
DMEM: Dulbecco's modified Eagle's medium
snRNA: small nuclear RNA
snoRNA: small nucleolar RNA
qRT: quantitative RT
SILAC: stable isotope labeling of amino acids in culture
BisTris: 2-[bis(2-hydroxyethyl)amino]-2-(hydroxymethyl)propane-1,3-diol
GAPDH: glyceraldehyde-3-phosphate dehydrogenase
OPC: oligodendrocyte precursor cell.
