# Supplementary material for: Leukodystrophy-associated POLR3A mutations down-regulate the RNA polymerase III transcript and important regulatory RNA BC200
Source: J Biol Chem. 2019 Mar 21;294(18):7445–59. doi: 10.1074/jbc.RA118.006271 (PMC6509492; doi:10.1074/jbc.RA118.006271)
Supplement: Supporting Information [file supp_294_18_7445__index.html]

Leukodystrophy-associated POLR3A mutations down-regulate the RNA polymerase III transcript and important regulatory RNA BC200 — RNA polymerase III mutations and leukodystrophy — Leukodystrophy-associated POLR3A mutations down-regulate the RNA polymerase III transcript and important regulatory RNA BC200 — RNA polymerase III mutations and leukodystrophy — Supporting Information 

# Leukodystrophy-associated *POLR3A* mutations down-regulate the RNA polymerase III transcript and important regulatory RNA *BC200*

## Supporting Information

- Supporting Information (to be published online) - Supplementary Tables
- Supporting Information (to be published online) - Supplementary Methods and Figures
