# Supplementary material for: Leukodystrophy-associated POLR3A mutations down-regulate the RNA polymerase III transcript and important regulatory RNA BC200
Source: J Biol Chem. 2019 Mar 21;294(18):7445–59. doi: 10.1074/jbc.RA118.006271 (PMC6509492; doi:10.1074/jbc.RA118.006271)
Supplement: Supporting Information [file supp_RA118.006271_141325_2_supp_296222_pnx8qd.pdf]

Supporting Information:

Selective effects of *POLR3A* leukodystrophy mutations on RNA Polymerase III transcripts

Supplementary Methods

CRISPR-Cas9 gene editing

For *POLR3A* mutant cell lines carrying the c.2554A>G (p.M852V) or c.2547C>G (p.F849L) mutations, we used CRISPR-Cas9 with homology-directed repair (HDR), as previously described.(1) Briefly, we selected two single guide RNAs (sgRNAs) targeting the region neighboring the c.2554A>G and c.2547C>G positions in *POLR3A* exon 19 using the CRISPR design tool http://crispr.mit.edu/. Oligonucleotides corresponding to both strands of the sgRNA sequences were annealed and cloned into the plasmid pSpCas9(BB)-2A-Puro (PX459) (#48139, Addgene) as previously described.(1) HDR templates containing the mutated nucleotide were synthesized (IDT) as single-strand DNA oligonucleotides (ssODN) with flanking homology arms of 40 or 90 nucleotides. Synonymous mutations in the PAM or sgRNA target sequences were also added to the ssODNs to prevent re-editing.(2) HEK293 or MO3.13 cells ($1x10^6$ cells per reaction) were re-suspended in 100µL of Ingenio® Electroporation Solution (Mirus Bio LLC) and nucleofected with the sgRNA-containing plasmid (2.5µg) and ssODN (75 pmols) using a Lonza-Amaxa® Nucleofector Device, then seeded into two wells of a 6-well plate. Forty-eight hours later, puromycin was added to the media at a concentration of 1µg/mL to select for cells having integrated the plasmid. Puromycin-resistant clonal cell colonies were subsequently screened using PCR amplification of *POLR3A* exon 19 followed by Sanger sequencing (McGill University and Genome Québec Innovation Center). Clones were considered positive if they carried the M852V or F849L mutation on all alleles (homozygous) or in compound heterozygosity with an indel causing a frameshift and premature stop codon. Clones that did not acquire mutations were used as controls for subsequent experiments. Three clones carrying the M852V mutation (M1 to M3) were used for all experiments in HEK293 cells and are described in the main text. One clone (M4) was homozygous for the F849L mutation and was used for Northern blots only. For compound heterozygous clones in MO3.13 cells,

PCR products were sequenced on an Illumina MiSeq (McGill University and Genome Québec Innovation Center) to confirm that the missense mutation and the deletion were on different alleles.

For *BC200* KO cell lines, we used a CRISPR-Cas9 approach adapted from ref (3), with dual sgRNAs targeting upstream and downstream of the *BC200* gene (Fig. S8a). We tested four different combinations of sgRNAs: g1+g3, g1+g4, g2+g3, g2+g4 (Table S10). sgRNA sequences were cloned into the plasmid pSpCas9(BB)-2A-Puro (PX459) as described above. Plasmids (2.5 μg) were transfected into MO3.13 cells with Lipofectamine 3000 and puromycin was added to the media at a concentration of 1μg/mL after forty-eight hours. Puromycin-resistant clonal cell colonies were screened by PCR for the presence of a band of approximately 200 bp corresponding to the targeted region without the *BC200* gene (Fig. S8b). To confirm complete deletion of the BC200 gene, PCR products were sequenced on an Illumina MiSeq (Fig. S8c). The resulting reads were trimmed with Trimmomatic(4) and aligned to the reference genome hg19 using STAR v2.3.0e(5). Aligned reads overlapping with the BC200 gene were identified and their cigar strings were parsed using Pybedtools(6) to determine if the gene was present (one or more mapped base in the BC200 gene) or deleted (no mapped base in the BC200 gene). We identified two clones with deletion of BC200 on all alleles (Fig. S8c) and absent BC200 RNA expression (Fig. S8d). All primers used for sgRNA cloning, PCR and sequencing are indicated in Table S10. For each sgRNA targeting *POLR3A* or *BC200*, we screened the top five possible off-target effects predicted by http://crispr.mit.edu/ by PCR and Sanger sequencing.

Small RNA and tRNA precursor sequencing

Since Pol III transcripts range in size from 70 to 330 nucleotides, we used two complementary RNA-seq approaches, consisting of rRNA-depleted RNA-seq for transcripts ≥ 200 nt and a modified small RNA-seq approach aimed at measuring the levels of Pol III transcripts < 200 nt, with a special focus on tRNA precursors (pre-tRNAs). Because of their short half-lives, pre-tRNAs have been shown to provide a more reliable estimate of Pol III transcription compared to mature tRNAs.(7-9) They are also easier to quantify by RNA-seq because they have not yet acquired the post-transcriptional modifications or complex

secondary structure that can interfere with reverse transcription. However, pre-tRNAs are only present at low coverage in standard RNA-seq data because of their size (~100 nt). Conversely, commercial small RNA-seq kits are often biased towards Dicer or Drosha-processed small RNAs and do not offer optimal coverage of Pol III transcripts. To overcome these limitations, we enriched total RNA extractions for small RNAs (< 200 nt), directly followed by random priming, cDNA synthesis and next-generation sequencing (Fig. S5a). Small RNA enrichments were performed using the miRNeasy kit (Qiagen) with the modifications outlined in Appendix A of the manufacturer's protocol to allow for separation of RNAs smaller than 200 nucleotides.

To monitor the level of small RNA enrichment in each sample, we synthesized three spike-in RNAs of different sizes (70, 94 and 250 nt, selected from previous publications), and added them at the beginning of the procedure. The two small spike-in RNAs were chosen for their similar size to that of Pol III transcripts: 70 nt for SS-70 in Locati *et al.(10)* and 94 nt for the synthetic spike-in from Zhong *et al.(11)* The larger 250 nt spike-in RNA corresponds to ERCC-00051 from the ERCC spike-in set,(12) but without the polyA tail. PCR products to be used for *in vitro* transcription reactions were generated using a G-block (IDT) template primer pairs corresponding to each spike-in RNA (Table S10). *In vitro* transcription was performed using PCR products as individual templates with a MAXIscript T7 *in vitro* Transcription Kit (Ambion). Completed reactions were treated with TurboDNase (Ambion) and subsequently loaded onto mini Quick Spin RNA Columns (Roche) to remove unincorporated nucleotides. RNA was phenol-chloroform extracted and analyzed by 7% denaturing PAGE. Full-length RNA molecules were then eluted from the gel and quantified by Nanodrop. Synthetic spike-in RNAs were mixed at an equimolar concentration of $4x10^{-9}$ mol/L and 0.5 µL of this mix was added to Qiazol lysis buffer after cells were homogenized.

Small RNA enrichment was confirmed using an Agilent Bioanalyzer (Fig. S5a). Libraries from three HEK293 mutant clones (M1-M3) and three control clones (C1-C3) were prepared with the KAPA

stranded RNA-seq library preparation and sequenced on an Illumina HiSeq 2500 with 100bp single-end reads. Since the three mutant clones have slightly different genotypes (Fig. 1a), we also sequenced small RNA-seq libraries from biological triplicates of the mutant clone with the lowest *POLR3A* expression (M2, see Fig. 1b to 1d) and a control clone (C3), in order to assess the impact of POLR3A hypofunction in the worst-case scenario.

Quality control and trimming were performed as previously described.(13) Trimmed reads were aligned to the reference genome hg19 using STAR v2.3.0e(5), including reads mapping to up to 100 locations. tRNA gene body coverage was assessed with RSeQC(14) using windows encompassing each tRNA gene and 20bp upstream and downstream. This showed good coverage of the tRNA gene body (Fig. S6b), with only a slight drop in coverage near the 3' end. Expression levels were estimated with featureCounts(15) using exonic reads in three successive runs with different parameters to treat multimapping reads: *i)* uniquely mapped reads only; *ii)* all multimapping reads, counting primary alignments only; and *iii)* all multimapping reads, counting primary and secondary alignments. All subsequent analyses were performed with the three types of counts. Unless otherwise specified, results are reported for option ii), but general agreement of results was verified with the three options. Expression levels of tRNA precursors were estimated by counting reads mapping at least partially to tRNA introns, leader (20bp upstream) or trailer (20bp downstream) sequences, using featureCounts(15) and custom scripts. pre-tRNA reads represented on average 74.9% of the total number of uniquely mapped tRNA reads and 37.8% of all mapped tRNA reads, while the remainder were exonic reads that could not distinguish between mature and pre-tRNAs (Fig. S6c). We used both types of reads for further analyses.

To assess small RNA enrichment, we calculated the ratio of counts from the small spike-ins over counts from the large spike-in. As a second measure of enrichment, we quantified the library size factors with DESeq2 for small (< 200 nt) and large RNAs (≥ 200 nt), respectively. The ratio of size factors was highly correlated to the ratio of spike-in counts (Fig. S6a), indicating that both measures can be used to assess

small RNA enrichment level. Thus, to account for small RNA enrichment variability during subsequent analyses, expression levels of small and large RNAs were normalized with their respective size factors, using DESeq2.(16) For transcripts with multiple isoforms, the maximum size was used. For small RNAs, tRNAs were excluded from the size factor calculation since they represent ~38% of expressed small transcripts and could thus skew the size factors if they are differentially expressed in mutants. After normalization, small and large transcripts were combined for the remainder of the differential expression analysis workflow with DESeq2. Differentially expressed genes were considered statistically significant if adjusted p-value (FDR) < 0.05 and mean expression >10.

Microarray

Total RNA was extracted from control and patient fibroblasts in triplicate using miRNeasy (Qiagen). LC Sciences (Houston, TX, USA) generated a custom microarray including three different probes (~22 nt) for each known Pol III transcript and pseudogene. Briefly, custom probes were synthesized on Paraflo® microfluidic chips. 5µg of total RNA was reverse transcribed and hybridized to the microarray in biological triplicates (LC Sciences). Following cross-array normalization of samples, probes with a mean intensity signal < 100 were excluded from analysis. Of the three probes targeting BC200 RNA, two had very low signal intensity and were excluded. The remaining probe, 5'-CGTAACTTCCCTCAAAGCAACAACCCC-3', targeted the unique 3' region of the transcript and showed a statistically significant difference between patients and controls.

SILAC

Cells were grown in DMEM for SILAC (ThermoFisher, #88364) supplemented with 10% dialyzed fetal bovine serum (ThermoFisher, #A3382001) and light [unlabeled lysine (ThermoFisher, #88429) and arginine (ThermoFisher, #89989)], medium [Lys4 (ThermoFisher, #88437) and Arg6 (ThermoFisher, #88210)] or heavy [Lys8 (ThermoFisher, #88209) and Arg10 (ThermoFisher, #89990)] amino acids. The three media were also supplemented with L-proline (ThermoFisher, # 88211). After > 6 passages in the

SILAC media, cells were harvested, washed in cold PBS and lysed in 1.5% n-dodecyl-β-D-maltoside (DDM) in PBS. Protein concentration was determined using DC Protein assay (Bio-Rad) and equal quantities of protein (25 or 30 μg) from the three conditions were mixed. Proteins were precipitated with methanol-chloroform and pellets were air-dried and stored at -80ºC. Protein extracts were re-solubilized in 6M urea buffer, reduced in reduction buffer (45 mM DTT, 100 mM ammonium bicarbonate) for 30 min at 37ºC, and then alkylated in alkylation buffer (100 mM iodoacetamide, 100 mM ammonium bicarbonate) for 20 min at 24ºC in dark. Prior to trypsin digestion, 50 mM ammonium bicarbonate was added to reduce the urea concentration under 2M. Proteins were digested with trypsin at 37ºC for 18 h and stopped with 5% formic acid. Protein digests were dried down in vacuum centrifuge and stored at -20ºC. Prior to LC-MS/MS, protein digests were re-solubilized under agitation for 15 min in 2%ACN / 1% formic acid and loaded into a 75 μm i.d. × 150 mm Self-Pack C18 column installed on the Easy-nLC II system (Proxeon Biosystems). Peptides were eluted with a two-slope gradient at a flowrate of 250 nL/min. Solvent B was first increased from 1 to 35% in 100 min and then from 35 to 84% B in 20 min. The HPLC system was coupled to Orbitrap Fusion mass spectrometer (Thermo Scientific) through a Nanospray Flex Ion Source. Nanospray and S-lens voltages were set to 1.3-1.8 kV and 50 V, respectively. Capillary temperature was set to 225°C. Full scan MS survey spectra (m/z 360-1560) in profile mode were acquired in the Orbitrap with a resolution of 120,000 and a target value at 1e6. The 25 most intense peptide ions were fragmented in the HCD collision cell and analyzed in the linear ion trap with a target value at 2e4 and a normalized collision energy at 28. Target ions selected for fragmentation were dynamically excluded for 25 sec.

Raw data files were processed with MaxQuant(17) v.1.6.0.16. MS/MS spectra were matched against the human Uniprot annotation (downloaded on November 11, 2017). Methionine oxidation was set as a variable modification and Lys4/Arg6 and Lys8/Arg10 were set as medium and heavy labels, respectively. Trypsin/P was defined as enzyme, allowing for two missed cleavages. FDR threshold was set to 0.01 for peptide and protein identifications. Minimal ratio count was set to 2 for protein quantification and the

functions “match between runs”, “requantify” and “match from and to” were enabled. Normalized MaxQuant ratios were used for subsequent analyses with Perseus v.1.5.6.0. Known protein contaminants were removed from the analysis. Protein groups were kept for analysis if they were detected in at least four out of six biological replicates. Conditions (POLR3A^{M852V} or BC200^{KO} vs. MO3.13-WT) were compared in pairs using a one-sample t-test on log2 ratios. Multiple testing correction was performed using the Benjamini-Hochberg method. The threshold for statistical significance was set at FDR < 0.05. Proteins were considered to be significantly differentially abundant when absolute log2 fold change (BC200^{KO}/WT or POLR3A^{M852V}/WT) > 0.5. Subsequent analyses were performed using custom scripts in R. We used the software GOrilla(18) for GO analysis, with all detected proteins as background.

For comparison of RNA-seq and SILAC data, mRNA/protein pairs were kept if they were properly expressed at the mRNA level (mean normalized expression across samples > 100) and were detected in at least four out of six SILAC replicates, in which peptide counts were assigned to only one protein. For identification of protein changes that could be the result of translational regulation in BC200^{KO}, we calculated Δ as the difference between the SILAC log2 fold change (log2FC$_S$) and the RNA-seq log2 fold change (log2FC$_R$). We computed the quartiles of Δ, log2FC$_S$ and log2FC$_R$:

**BC200-KO**	**0%**	**25%**	**50%**	**75%**	**100%**
Δ	0.00011	0.0960	0.2059	0.3697	2.4055
log2FC$_S$	0.00038	0.1116	0.2604	0.4722	3.0099
log2FC$_R$	0.00025	0.1073	0.2334	0.4248	2.6194

We considered proteins to undergo protein-level changes only if SILAC FDR < 0.05, Δ was in the upper quantile, the absolute log2FC$_S$ was in the upper quantile and the absolute log2FC$_R$ was below the median (Fig. S11). We considered proteins to undergo substantially greater protein-level than mRNA-level changes if they did not belong to the above category, SILAC FDR < 0.05, Δ was in the upper quantile and the absolute log2FC$_S$ was in the upper quantile (Fig. S11). Finally, we considered proteins to be

regulated at the mRNA level when SILAC FDR < 0.05, RNA-seq adjusted p-value < 0.05, the absolute $log2FC_R$ was in the upper quartile and Δ was smaller than the tercile.

Supplementary Figures

a

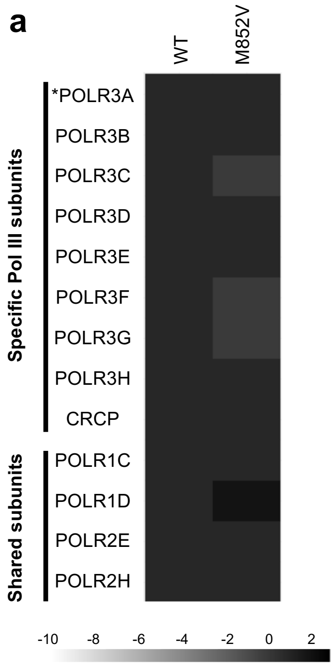

Protein Symbol	average POLR3A-WT	average POLR3A-M852V	ratio WT/POLR3A-M852V	pvalue	adjusted pvalue
POLR3A	256	256	1	1	1
POLR3B	121.258	116.91	0.964	0.935	0.997
POLR3C	115.804	52.304	0.452	0.471	0.902
POLR3D	37.034	18.642	0.503	0.536	0.902
POLR3E	98.196	89.207	0.908	0.724	0.902
POLR3F	65.375	20.302	0.311	0.403	0.902
POLR3G	28.43	10.241	0.36	0.213	0.902
POLR3H	23.684	25.042	1.057	0.814	0.902
CRCP	25.148	18.361	0.73	0.577	0.902
POLR1C	44.086	50.464	1.145	0.672	0.902
POLR1D	3.948	8.16	2.067	0.467	0.902
POLR2E	24.349	20.922	0.859	0.748	0.902
POLR2H	12.817	10.361	0.808	0.617	0.902

b

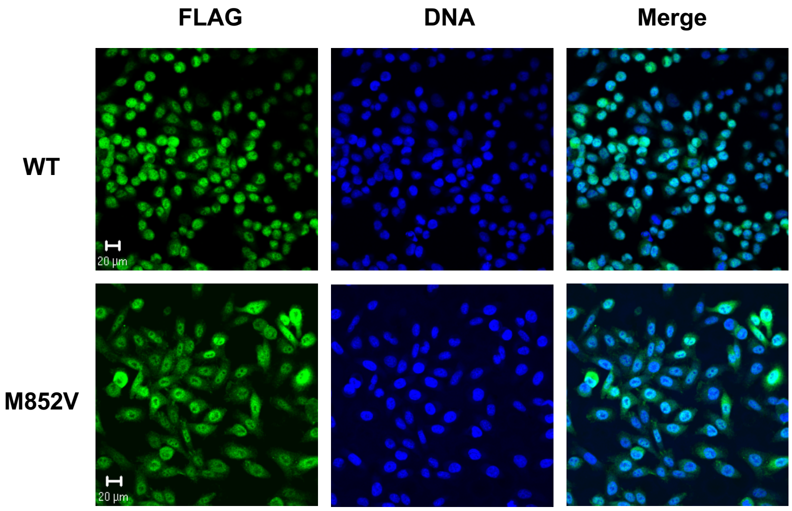

Figure S1. The POLR3A M852V mutation does not affect Pol III complex assembly. a) FLAG-tagged variants of POLR3A (WT or M852V) were expressed at equivalent levels in HeLa cells and purified using anti-FLAG affinity chromatography. The co-purified proteins were identified by LC-MS/MS. The heatmap contains the log2-transformed average spectral count ratios of mutant/WT across both replicates. Specific and shared (with Pol I and/or Pol II) subunits are identified on the left. POLR3A (the bait) is identified by an asterisk. The table contains average spectral counts across duplicates for each condition. The p-values and adjusted p-values were obtained by performing a two-tailed one-sample t-test and by multiple testing correction using the Benjamini-Hochberg procedure. Spectral counts were computed with Mascot. b) Immunofluorescence experiment showing the subcellular localization of FLAG-tagged variants of POLR3A. Scale bar = 20µm.

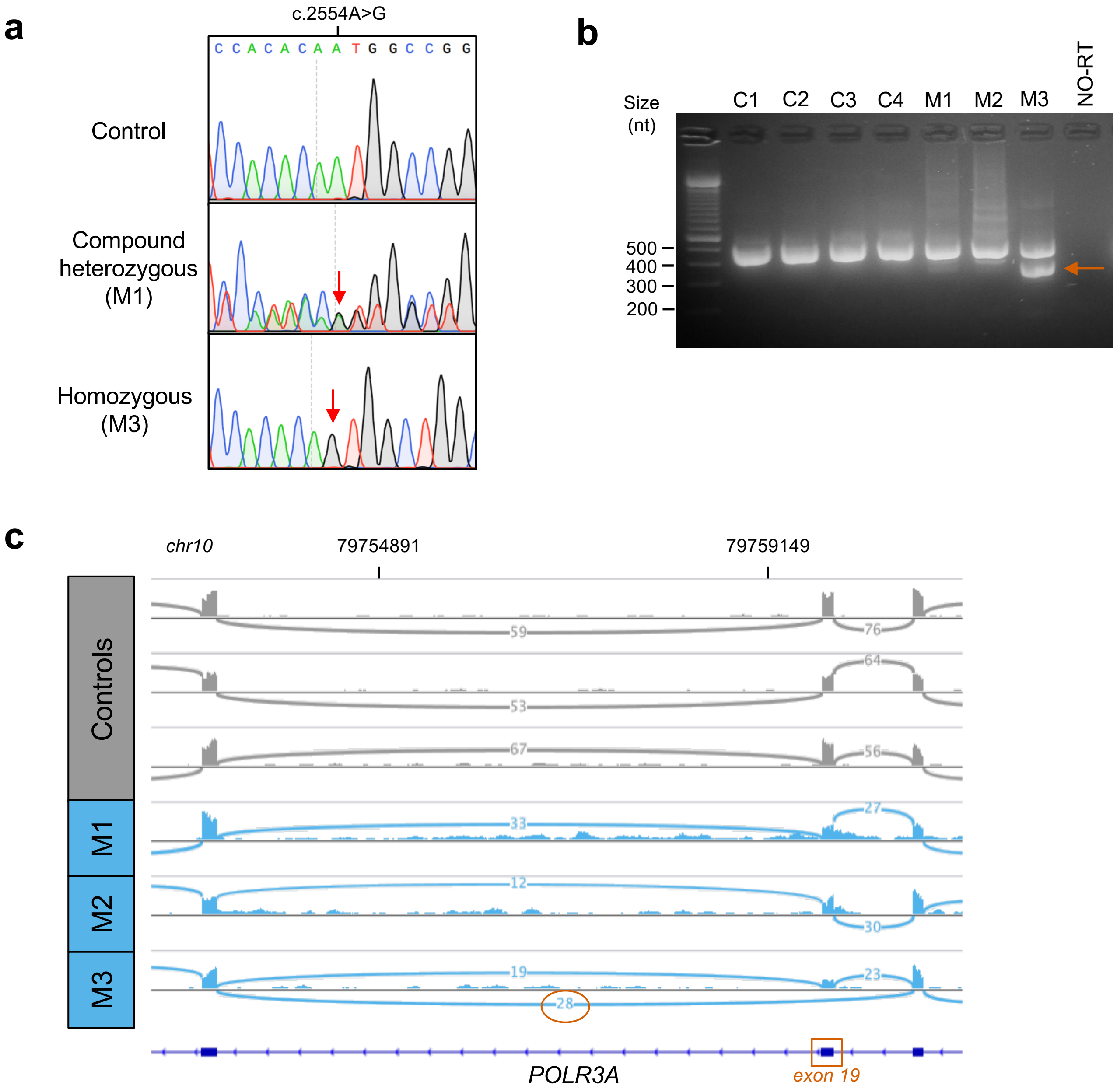

Figure S2. Characterization of *POLR3A* mutant clones generated by CRISPR-Cas9. a) Genomic DNA sequence chromatograms of control and *POLR3A* mutant clones. Red arrows indicate the mutation of interest (*POLR3A* c.2554A>G). As shown by the double sequence, compound heterozygous clones also carry an indel causing a frameshift and a premature stop codon. b) Electrophoresis of the PCR product from the amplification of exons 17-21 of *POLR3A* cDNA in four control clones (C1-C4) and three *POLR3A* mutant clones (M1-M3) in HEK293 cells. Mutant M3 shows a lower band corresponding to exon 19 skipping (orange arrow). c) Sashimi plot showing partial exon 19 skipping in mutant M3 (highlighted by the orange oval), while it has 100% inclusion in other mutants and controls. Exon 19 is where the M852V mutation is located.

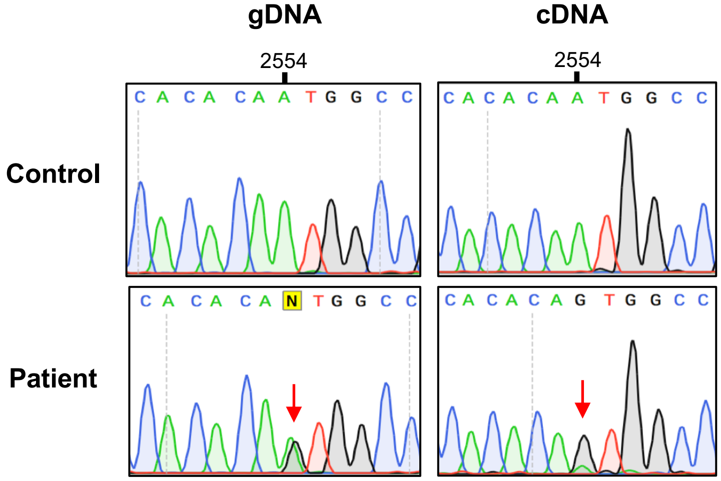

Figure S3. Expression of the *POLR3A* c.2554A>G mutation in a POLR3-HLD patient. gDNA and cDNA sequence chromatograms of a control and a patient carrying the c.2554A>G mutation in compound heterozygosity with a null allele. Both alleles are visible in the gDNA, while the missense allele is predominant in the cDNA, suggesting that the null allele is degraded.

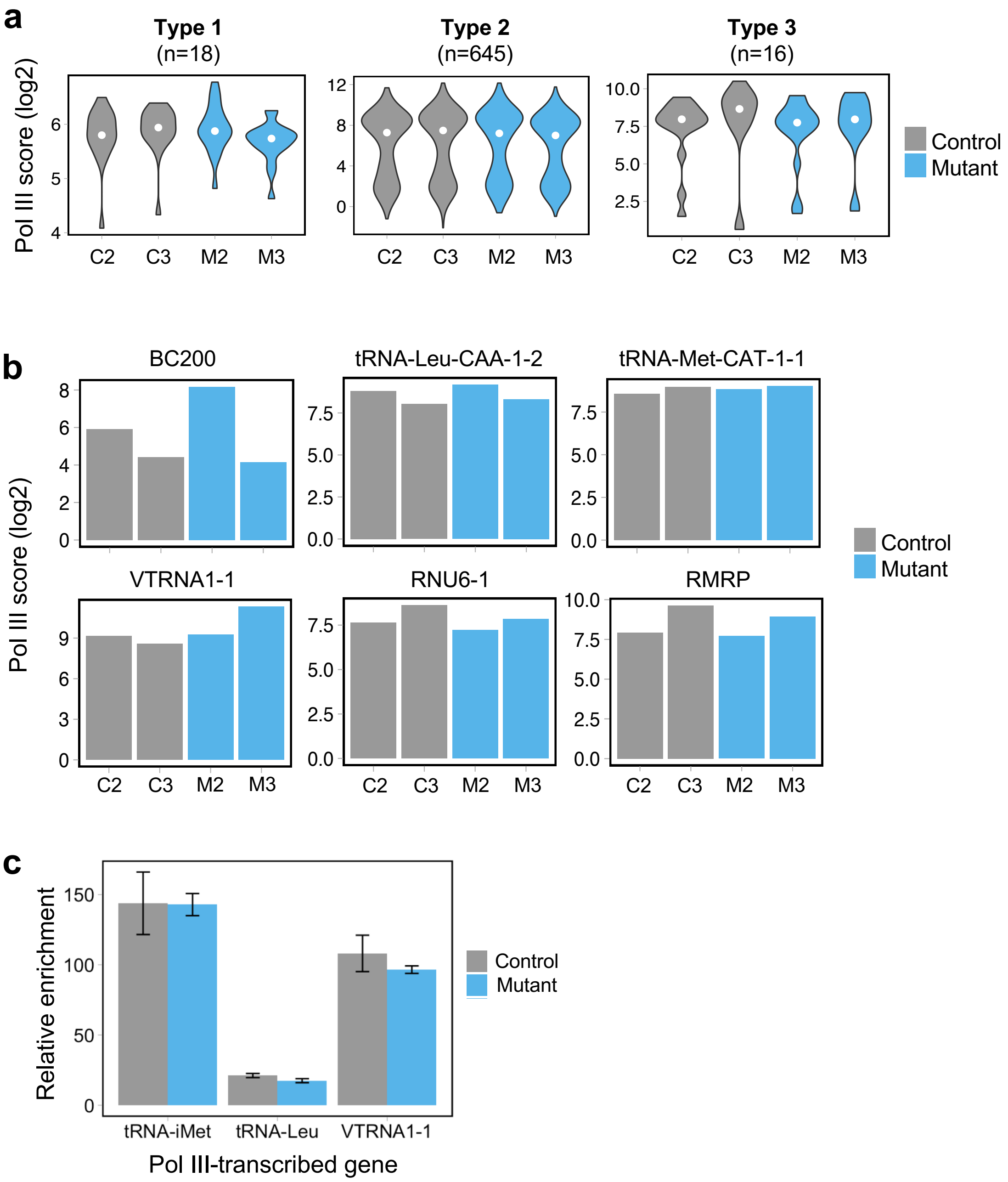

Figure S4. Pol III occupancy levels in control and *POLR3A* mutant HEK293 cells. a) Distribution of Pol III occupancy score measured by ChIP-seq against POLR3A in each sample for the three types of known Pol III-transcribed genes. The white dot indicates the median. b) Pol III occupancy score measured by ChIP-seq for POLR3A for six Pol III-transcribed genes. c) ChIP-qPCR performed against POLR3A. The chromatin was quantified by qPCR with primers for three Pol III target genes. Pol III enrichment at these loci was calculated relative to a locus on chromosome 13 that is not bound by Pol III. Data are represented as mean +/− SEM of biological replicates.

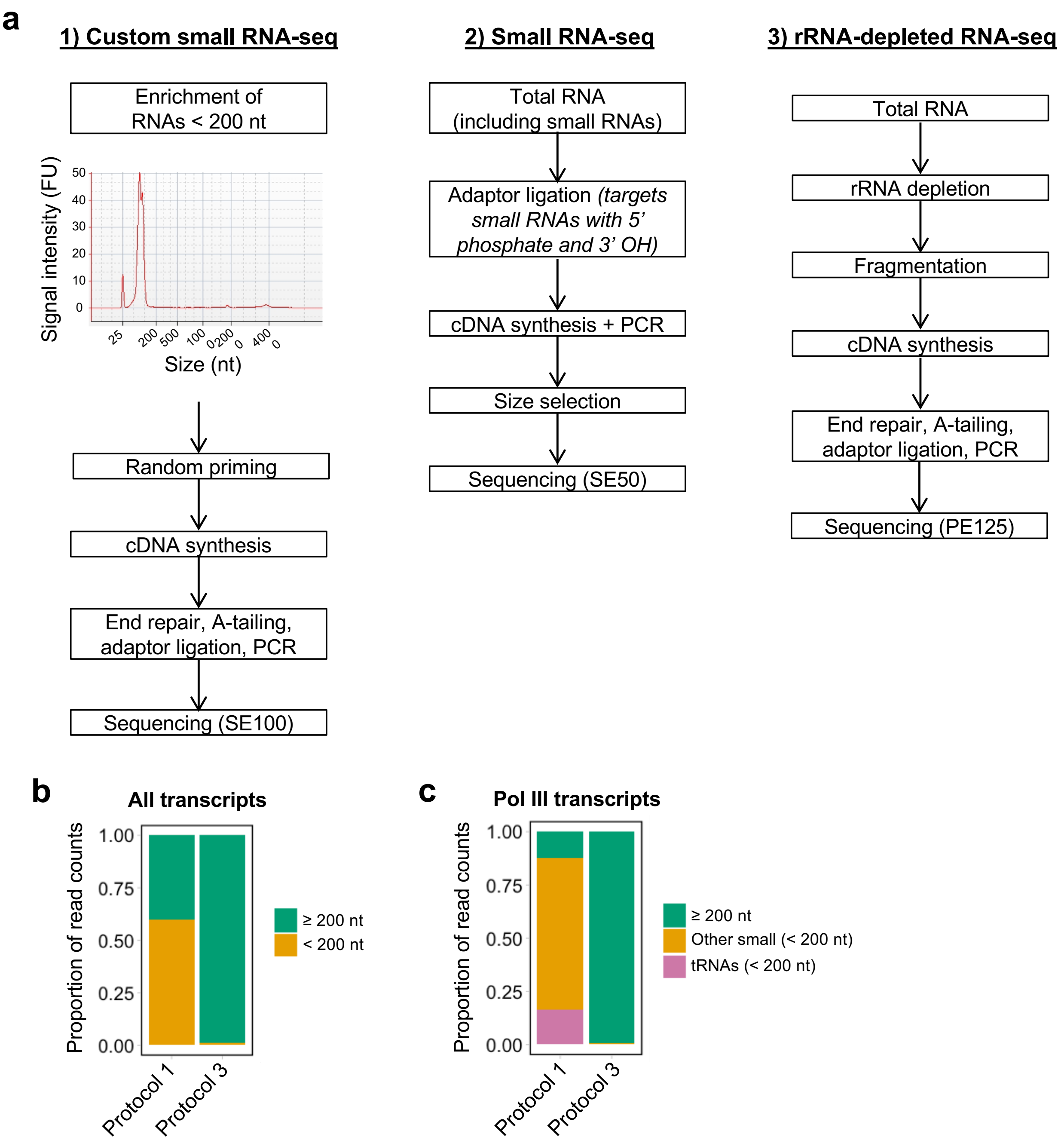

Figure S5. Optimization of a small RNA-seq approach for Pol III transcripts. a) Overview of the workflow for the custom small RNA-seq protocol used in this study compared to traditional small RNA-seq and rRNA-depleted RNA-seq approaches. b) Proportions of read counts mapping to small and large transcripts using custom small RNA-seq (protocol 1) compared to rRNA-depleted RNA-seq (protocol 3). c) Proportion of reads counts mapping to tRNAs, other small Pol III transcripts or large Pol III transcripts in the two protocols. b) and c) show an enrichment of small transcripts in Protocol 1 compared to Protocol 3.

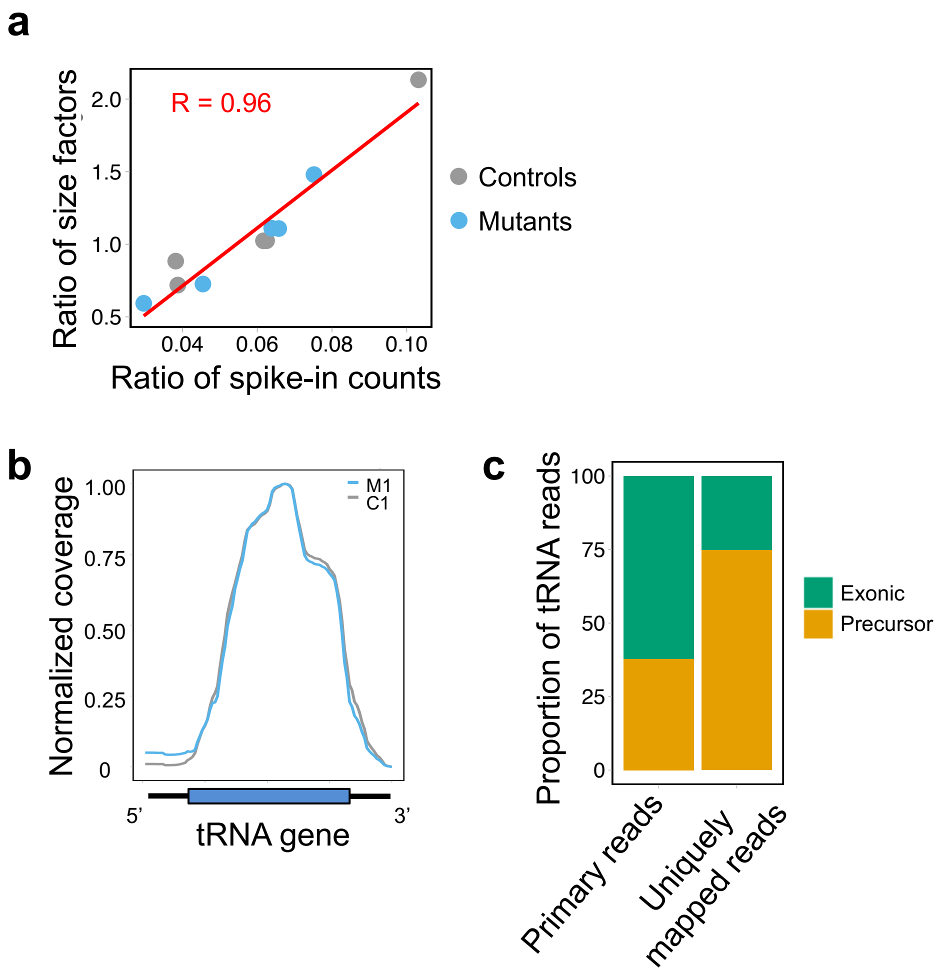

Figure S6. Analysis of small RNA-seq data. a) Correlation between the ratio of spike-in counts and the ratio of DESeq2 size factors calculated for small (< 200 nt) and large (≥ 200 nt) transcripts. b) Representative image of the aggregate coverage at all tRNA gene bodies in samples M1 and C1. Coverage was computed with RSeqQC for each tRNA gene, with 20bp upstream and downstream. c) Proportion of tRNA reads mapping to precursor or exonic regions of tRNAs, using all reads (each read counted once only) or uniquely mapped reads.

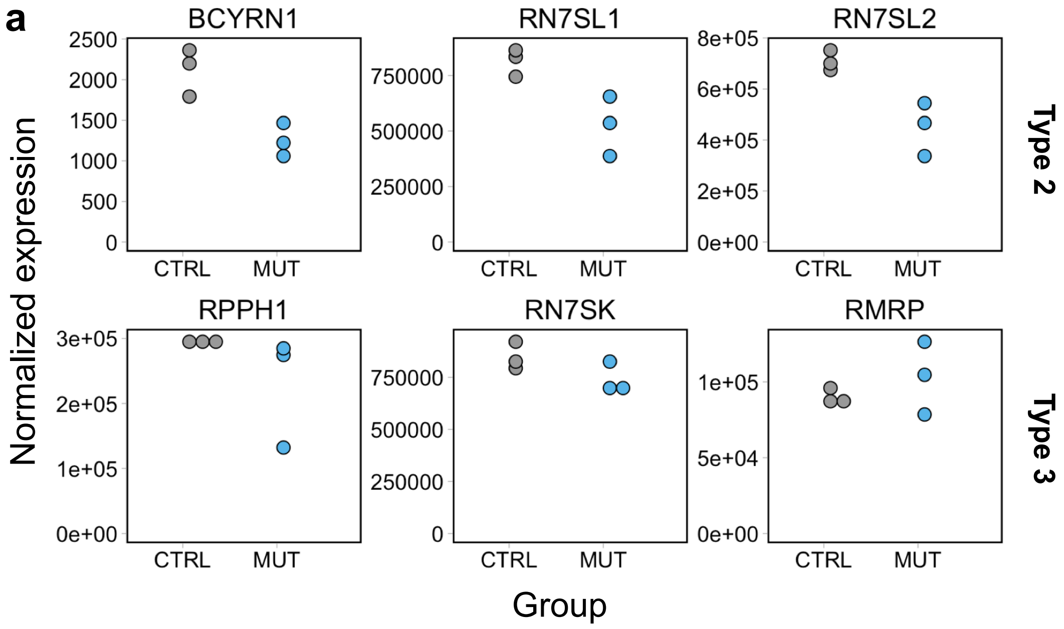

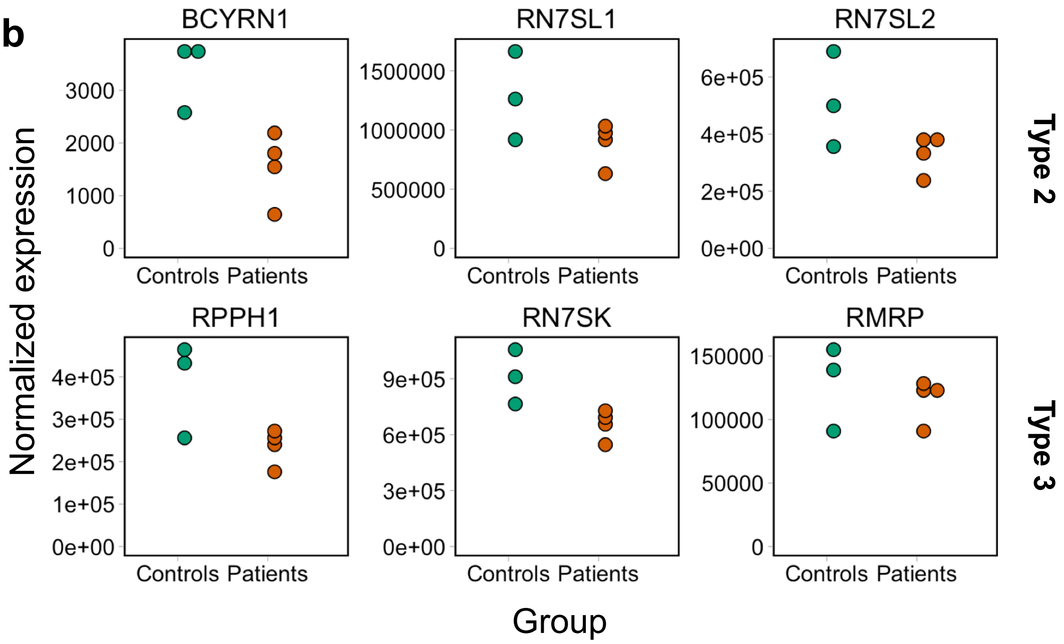

Figure S7. Expression of large Pol III transcripts in a) HEK293 control (CTRL) and mutant (MUT) clones and b) primary fibroblasts derived from controls and French Canadian POLR3-HLD patients. In both datasets, BCYRN1 (encoding BC200 RNA) shows the clearest difference in expression between normal and POLR3A-mutated samples. Pol III transcripts with type 2 internal elements are on the top row of each panel, and type 3 Pol III transcripts are on the bottom row. Normalized expression was computed with DESeq2.

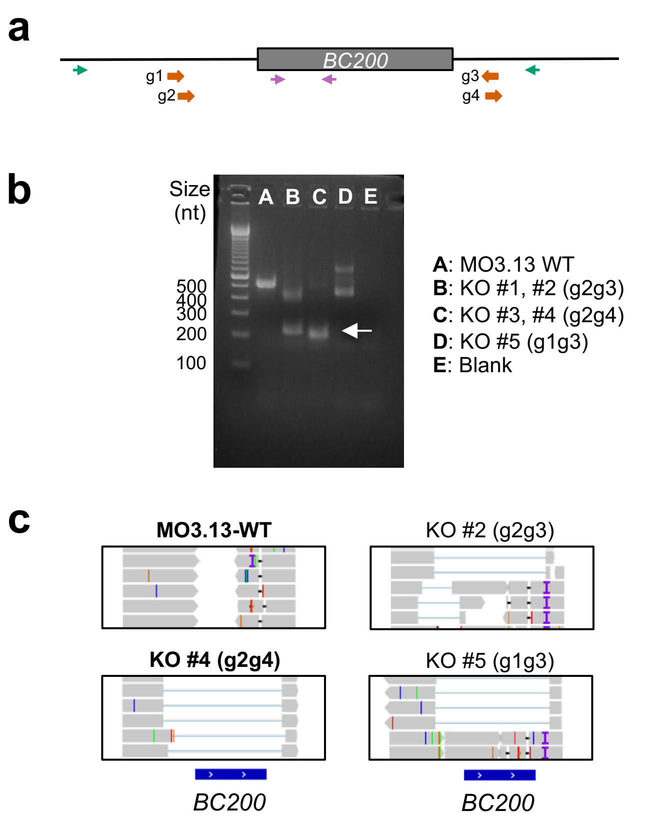

Sample	Reads with deletion	Reads without deletion	Percent with deletion (%)
MO3.13-WT	20	2672	0.74
**KO #4**	**5962**	**0**	**100.00**
KO #2	6640	916	87.88
KO #5	227	3317	6.41

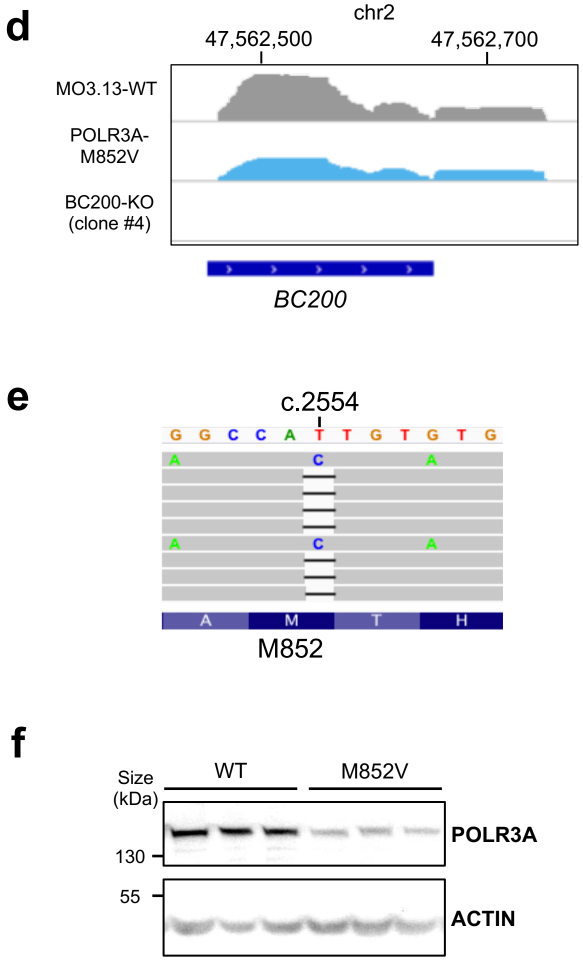

Figure S8. Generation of BC200^{KO} and POLR3A^{M852V} MO3.13 cell lines a) Schematic representation of the sgRNAs used to knock-out BC200 RNA. sgRNAs are shown in orange, primers for deletion screening are in green and qRT-PCR primers are in pink. sgRNAs were used in the following combinations: g1g3, g1g4, g2g3 and g2g4. b) Example of PCR products obtained in single clones obtained with each sgRNA combination using the deletion screening primers. The g1g4 combination only resulted in WT clones. The full-size PCR product including BC200 RNA is shown in sample MO3.13 WT. The arrow indicates the expected PCR product without BC200 RNA. c) Top: IGV view of representative reads obtained from MiSeq sequencing of the PCR products obtained in d). Bottom: Quantification of the number of reads with and without deletion of the entire BC200 gene. Only clone #4 displays a complete deletion of the BC200 gene. d) IGV view of the expression of BC200 RNA measured by RNA-seq in the parental MO3.13, POLR3A-M852V and BC200-KO (clone KO #4) cell lines, showing complete absence of expression in the BC200-KO clone. All samples are represented on the same scale (0-361). e) IGV view of the gDNA sequence of an MO3.13 *POLR3A* mutant clone surrounding the c.2554A>G position, obtained by MiSeq, showing compound heterozygosity for the M852V mutation and a one bp deletion causing a frameshift and premature stop codon. f) POLR3A protein levels in triplicates of the MO3.13 parental and POLR3A-M852V cell lines. ACTIN was used as a loading control.

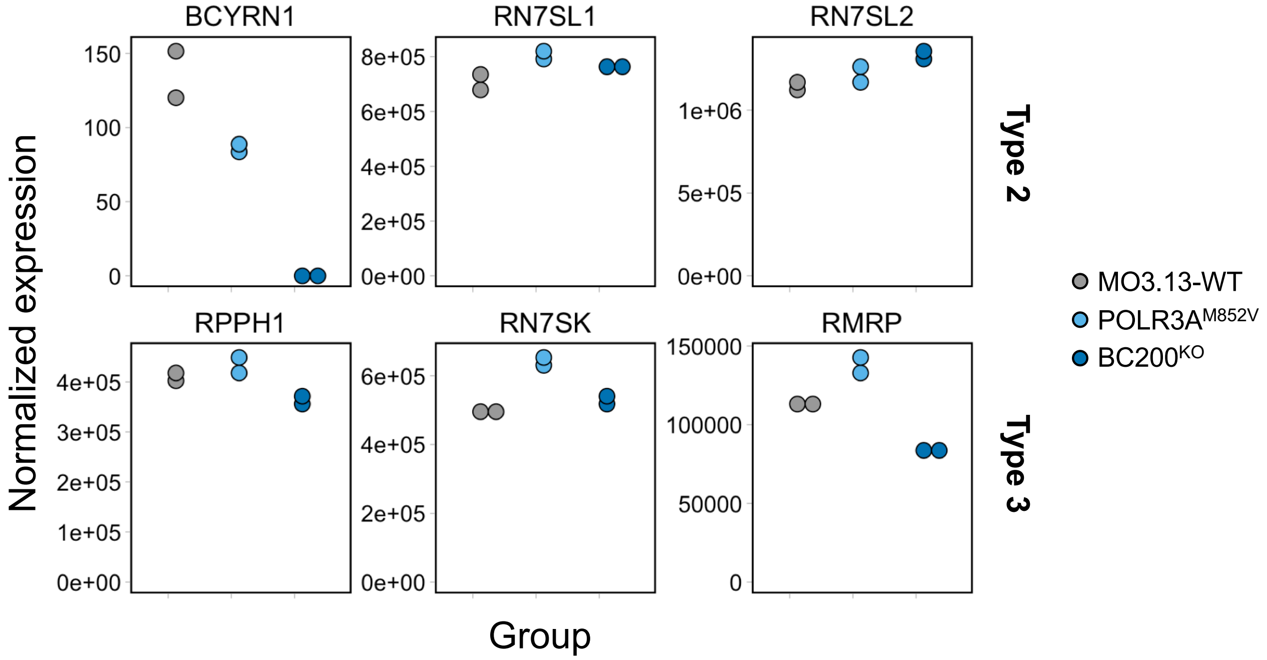

Figure S9. Expression of large Pol III transcripts in MO3.13-WT, POLR3A^{M852V} and BC200^{KO} cells, showing decreased expression of BCYRN1 (encoding BC200 RNA) in POLR3A^{M852V} compared to WT, but not of other Pol III transcripts. Type 2 Pol III transcripts are on the top row of each panel, and type 3 Pol III transcripts are on the bottom row.

a

Mutant vs. WT	SILAC		RNA-seq	
	|log2 FC| > 0.5	|log2 FC| > 1	|log2 FC| > 0.5	|log2 FC| > 1
POLR3A^{M852V}	93	7	1773	328
BC200^{KO}	276	60	3165	723

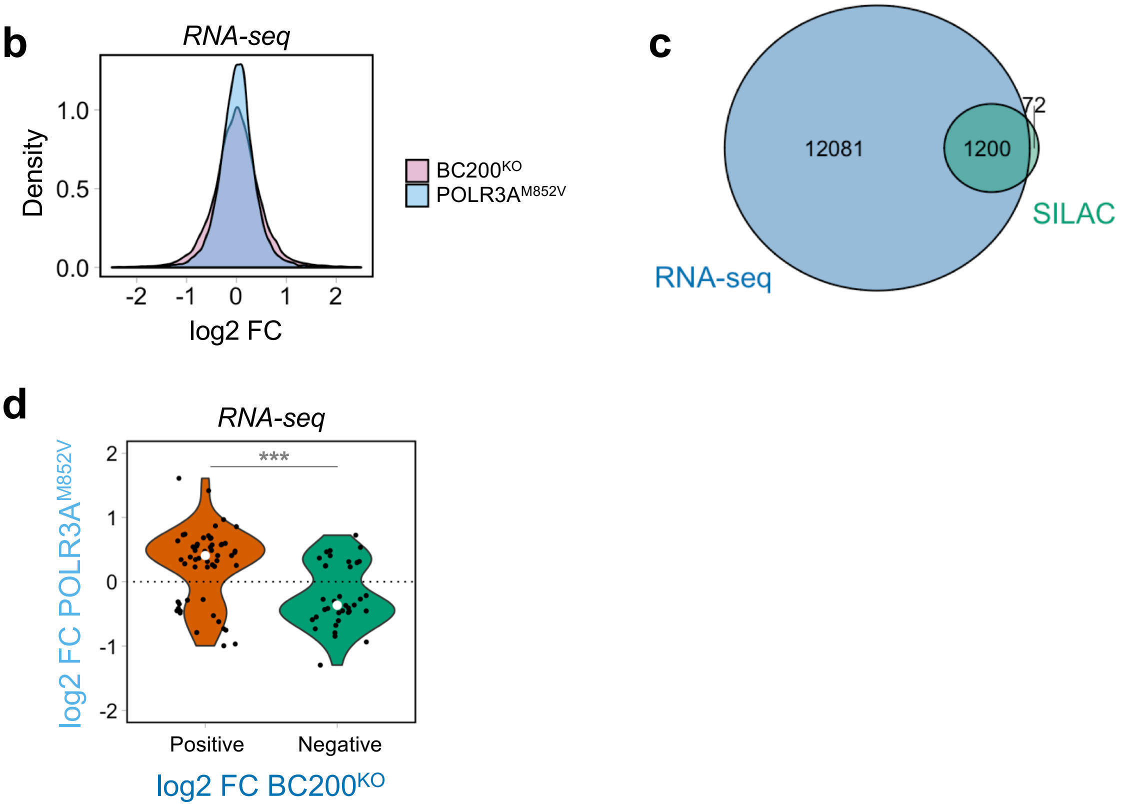

Figure S10. Impact of POLR3A mutation and BC200 KO on the transcriptome and proteome of MO3.13 cells. a) Number of differentially abundant proteins (SILAC) or mRNAs (RNA-seq) detected in each mutant compared to WT MO3.13 cells. Proteins were considered differentially abundant when FDR < 0.05 and absolute log2 fold change > 0.5 or 1. mRNAs were considered differentially expressed when the mean normalized expression across samples > 100, adjusted p-value < 0.05 and log2 fold change > 0.5 or 1. b) Distribution of the log2 fold change for POLR3A^{M852V}/WT and BC200^{KO}/WT using all expressed mRNAs (mean expression > 100) in RNA-seq data. More mRNAs have high fold changes in BC200^{KO} (p-value < 2.2 x 10^{-16}, two-sample Kolmogorov-Smirnov test). c) Overlap between mRNAs detected by RNA-seq (normalized expression > 100) and corresponding proteins detected by SILAC (in 4 out of 6 replicates). The 1,200 overlapping mRNA/protein pairs were used for subsequent analyses. d) Distribution of fold change in POLR3A^{M852V} for mRNAs that showed statistically significant differences in RNA-seq in both conditions (adjusted p-value < 0.05) and had a significant fold change in BC200^{KO} (log2 FC > 0.5). Only mRNAs also detected in SILAC were used for this analysis. ***p < 0.001, Wilcoxon rank-sum test.

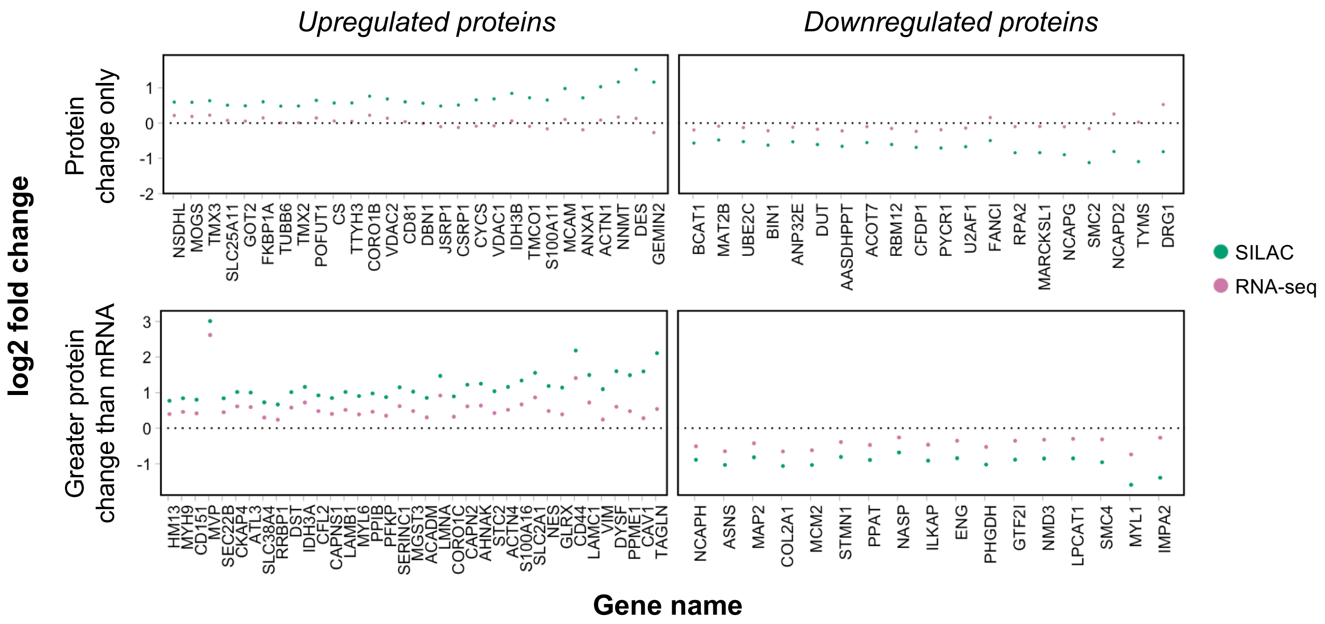

Figure S11. Identification of proteins regulated at the translational level. log2 fold change of BC200^{KO} vs. WT in SILAC and RNA-seq for mRNA/protein pairs where only the protein shows significant changes (top) or the protein shows substantially greater changes than the mRNA (bottom). The thresholds used for identifying these proteins are described in Supplementary Methods.

References

1. Ran, F. A., Hsu, P. D., Wright, J., Agarwala, V., Scott, D. A., and Zhang, F. (2013) Genome engineering using the CRISPR-Cas9 system. *Nature protocols* **8**, 2281-2308
2. Paquet, D., Kwart, D., Chen, A., Sproul, A., Jacob, S., Teo, S., Olsen, K. M., Gregg, A., Noggle, S., and Tessier-Lavigne, M. (2016) Efficient introduction of specific homozygous and heterozygous mutations using CRISPR/Cas9. *Nature* **533**, 125-129
3. Singh, R., Gupta, S. C., Peng, W. X., Zhou, N., Pochampally, R., Atfi, A., Watabe, K., Lu, Z., and Mo, Y. Y. (2016) Regulation of alternative splicing of Bcl-x by BC200 contributes to breast cancer pathogenesis. *Cell death & disease* **7**, e2262
4. Bolger, A. M., Lohse, M., and Usadel, B. (2014) Trimmomatic: a flexible trimmer for Illumina sequence data. *Bioinformatics* **30**, 2114-2120
5. Dobin, A., Davis, C. A., Schlesinger, F., Drenkow, J., Zaleski, C., Jha, S., Batut, P., Chaisson, M., and Gingeras, T. R. (2013) STAR: ultrafast universal RNA-seq aligner. *Bioinformatics* **29**, 15-21
6. Dale, R. K., Pedersen, B. S., and Quinlan, A. R. (2011) Pybedtools: a flexible Python library for manipulating genomic datasets and annotations. *Bioinformatics* **27**, 3423-3424
7. Bonhoure, N., Byrnes, A., Moir, R. D., Hodroj, W., Preitner, F., Praz, V., Marcelin, G., Chua, S. C., Jr., Martinez-Lopez, N., Singh, R., Moullan, N., Auwerx, J., Willemin, G., Shah, H., Hartil, K., Vaitheesvaran, B., Kurland, I., Hernandez, N., and Willis, I. M. (2015) Loss of the RNA polymerase III repressor MAF1 confers obesity resistance. *Genes & development* **29**, 934-947
8. Upadhya, R., Lee, J., and Willis, I. M. (2002) Maf1 is an essential mediator of diverse signals that repress RNA polymerase III transcription. *Molecular cell* **10**, 1489-1494
9. Orioli, A., Praz, V., Lhote, P., and Hernandez, N. (2016) Human MAF1 targets and represses active RNA polymerase III genes by preventing recruitment rather than inducing long-term transcriptional arrest. *Genome research*
10. Locati, M. D., Terpstra, I., de Leeuw, W. C., Kuzak, M., Rauwerda, H., Ensink, W. A., van Leeuwen, S., Nehrdich, U., Spaink, H. P., Jonker, M. J., Breit, T. M., and Dekker, R. J. (2015) Improving small RNA-seq by using a synthetic spike-in set for size-range quality control together with a set for data normalization. *Nucleic acids research* **43**, e89
11. Zhong, J., Xiao, C., Gu, W., Du, G., Sun, X., He, Q. Y., and Zhang, G. (2015) Transfer RNAs Mediate the Rapid Adaptation of Escherichia coli to Oxidative Stress. *PLoS genetics* **11**, e1005302
12. Baker, S. C., Bauer, S. R., Beyer, R. P., Brenton, J. D., Bromley, B., Burrill, J., Causton, H., Conley, M. P., Elespuru, R., Fero, M., Foy, C., Fuscoe, J., Gao, X., Gerhold, D. L., Gilles, P., Goodsaid, F., Guo, X., Hackett, J., Hockett, R. D., Ikonomi, P., Irizarry, R. A., Kawasaki, E. S., Kaysser-Kranich, T., Kerr, K., Kiser, G., Koch, W. H., Lee, K. Y., Liu, C., Liu, Z. L., Lucas, A., Manohar, C. F., Miyada, G., Modrusan, Z., Parkes, H., Puri, R. K., Reid, L., Ryder, T. B., Salit, M., Samaha, R. R., Scherf, U., Sendera, T. J., Setterquist, R. A., Shi, L., Shippy, R., Soriano, J. V., Wagar, E. A., Warrington, J. A., Williams, M., Wilmer, F., Wilson, M., Wolber, P. K., Wu, X., Zadro, R., and External, R. N. A. C. C. (2005) The External RNA Controls Consortium: a progress report. *Nature methods* **2**, 731-734
13. Antonicka, H., Choquet, K., Lin, Z. Y., Gingras, A. C., Kleinman, C. L., and Shoubridge, E. A. (2017) A pseudouridine synthase module is essential for mitochondrial protein synthesis and cell viability. *EMBO Rep* **18**, 28-38
14. Wang, L., Wang, S., and Li, W. (2012) RSeQC: quality control of RNA-seq experiments. *Bioinformatics* **28**, 2184-2185
15. Liao, Y., Smyth, G. K., and Shi, W. (2014) featureCounts: an efficient general purpose program for assigning sequence reads to genomic features. *Bioinformatics* **30**, 923-930
16. Love, M. I., Huber, W., and Anders, S. (2014) Moderated estimation of fold change and dispersion for RNA-seq data with DESeq2. *Genome biology* **15**, 550

17. Cox, J., and Mann, M. (2008) MaxQuant enables high peptide identification rates, individualized p.p.b.-range mass accuracies and proteome-wide protein quantification. *Nature biotechnology* **26**, 1367-1372
18. Eden, E., Navon, R., Steinfeld, I., Lipson, D., and Yakhini, Z. (2009) GOrilla: a tool for discovery and visualization of enriched GO terms in ranked gene lists. *BMC bioinformatics* **10**, 48
